# Disordered Expression of *shaggy*, the *Drosophila* Gene Encoding a Serine-Threonine Protein Kinase GSK3, Affects the Lifespan in a Transcript-, Stage-, and Tissue-Specific Manner

**DOI:** 10.3390/ijms20092200

**Published:** 2019-05-04

**Authors:** Mikhail V. Trostnikov, Natalia V. Roshina, Stepan V. Boldyrev, Ekaterina R. Veselkina, Andrey A. Zhuikov, Anna V. Krementsova, Elena G. Pasyukova

**Affiliations:** 1 Institute of Molecular Genetics of RAS, Kurchatov Sq. 2, 123182 Moscow, Russia; mikhail.trostnikov@gmail.com (M.V.T.); nwumr@yandex.ru (N.V.R.); beibaraban34@gmail.com (S.V.B.); veselkinaer@gmail.com (E.R.V.); homkabrut@gmail.com (A.A.Z.); akrementsova@mail.ru (A.V.K.); 2 Vavilov Institute of General Genetics, Russian Academy of Sciences, Gubkin 3, 119991 Moscow, Russia; 3 Emmanuel Institute of Biochemical Physics, Russian Academy of Sciences, Kosygin St. 4, 119334 Moscow, Russia

**Keywords:** GSK3 (glycogen synthase kinase 3), protein kinases, transcription, lifespan, the nervous system, *Drosophila melanogaster*

## Abstract

GSK3 (glycogen synthase kinase 3) is a conserved protein kinase governing numerous regulatory pathways. In *Drosophila melanogaster*, GSK3 is encoded by *shaggy* (*sgg*), which forms 17 annotated transcripts corresponding to 10 protein isoforms. Our goal was to demonstrate how differential *sgg* transcription affects lifespan, which GSK3 isoforms are important for the nervous system, and which changes in the nervous system accompany accelerated aging. Overexpression of three *sgg* transcripts affected the lifespan in a stage- and tissue-specific way: *sgg-RA* and *sgg-RO* affected the lifespan only when overexpressed in muscles and in embryos, respectively; the essential *sgg-RB* transcript affected lifespan when overexpressed in all tissues tested. In the nervous system, only *sgg-RB* overexpression affected lifespan, causing accelerated aging in a neuron-specific way, with the strongest effects in dopaminergic neurons and the weakest effects in GABAergic neurons. Pan-neuronal *sgg*-*RB* overexpression violated the properties of the nervous system, including the integrity of neuron bodies; the number, distribution, and structure of mitochondria; cytoskeletal characteristics; and synaptic activity. Such changes observed in young individuals indicated premature aging of their nervous system, which paralleled a decline in survival. Our findings demonstrated the key role of GSK3 in ensuring the link between the pathology of neurons and lifespan.

## 1. Introduction

GSK3 (glycogen synthase kinase 3) is an actively studied, highly conserved serine–threonine protein kinase that is primarily regulated by inhibition and is involved in multiple signaling pathways that regulate embryogenesis and differentiation; cell renewal, migration, and apoptosis; gene transcription; metabolism; and survival via more than 50 target proteins with phosphorylation sites for GSK3 [1,2]. In humans, there are two main forms of GSK3, α and β, encoded by the two highly homologous genes; in *Drosophila*, only one form exists, which is closer to GSK3β.

In *D. melanogaster*, GSK3 plays multiple roles in the development and function of the nervous system, starting from the early development stages. GSK3 directly phosphorylates aPKC (atypical protein kinase C) [3,4], a key component ensuring asymmetric neuroblast division during *D. melanogaster* early development. GSK3 participates in the development of neural precursor cells [5] and, via InR/TOR (Insulin Receptor/Target of Rapamycin) signaling, in the control of dendrite pruning during larval-pupal transition [6]. Moreover, GSK3 controls synapse formation, growth, and morphological structure by controlling the dynamics of the microtubule cytoskeleton [7,8,9] and regulates axonal stability and neural circuit integrity via the Wnt pathway [10]. GSK3 also regulates neurotransmitter release into NMJs (neuromuscular junctions) [8]. GSK3 directly phosphorylates microtubule-associated protein tau [11,12]. Overexpression of human tau in *D. melanogaster* disrupts axonal transport causing vesicle aggregation, and co-overexpression of constitutively active GSK3 enhances the effects [13]. Two functions of GSK3 specifically attracted our attention to this protein.

First, GSK3 participates in the control of asymmetric neuroblast division. Earlier, we demonstrated that functional changes in several genes involved in the regulation of this developmental process, namely, *aPKC* encoding the GSK3 target, aPKC, *escargot* and *inscutable*, affect the lifespan of adult flies [14,15]. Moreover, our previous studies suggest that changes in gene expression at the embryonic stage can be responsible for an increase in adult lifespan due to yet unknown mechanisms that provide this carry-over effect [16]. Therefore, we were interested in understanding whether GSK3 function, especially during the embryonic stage, also affects lifespan.

Second, GSK3 severely affects the functionality of the tau protein, which leads to the development of various age-dependent neurodegenerative diseases and significantly reduces life expectancy [11,17]. We were interested in understanding whether changes in GSK3 expression in the nervous system directly affect lifespan and, if yes, we aimed to identify which structural and functional properties of the nervous system accompany alterations in lifespan and rate of aging.

In *Drosophila melanogaster*, GSK3 is encoded by the gene, *shaggy* (*sgg*), which forms 17 annotated transcripts corresponding to 10 annotated protein isoforms (Available online: http://flybase.org/reports/FBgn0003371). To address the questions raised, first, it was necessary to understand which transcripts and protein isoforms encoded by *sgg* are functional. Ten *sgg* transcripts and at least five Sgg proteins of different primary structure were initially demonstrated [18]. The major Sgg isoform that is detected at essentially all stages of the life cycle and in different tissues [18,19] is encoded by six transcripts annotated in FlyBase (Available online: http://flybase.org/reports/FBgn0003371), including *sgg-RB* (GenBank #AY122193.1). Only a few other *sgg* transcripts were reported as full-size cDNAs: *sgg-RA* (GenBank #X70863.1); *sgg-RD* (GenBank #BT133153.1); *sgg-RG* (GenBank #AY119664.1 and #BT050474.1); and *sgg-RO* (GenBank #BT072831.1). Analysis of the proteome revealed three Sgg isoforms, namely, Sgg-PA, Sgg-PB, and Sgg-PD, in the embryonic cell line, Kc167; no isoforms were found in fly tissues [20,21,22]. Two isoforms, Sgg-PA and Sgg-PB, corresponding to sgg39 and sgg10 isoforms of Ruel and coauthors [18], were detected in recent functional studies; however, the functionality of alterations in Sgg-PA expression was not revealed [9,23].

In this paper, we described the effects of the misexpression of four *sgg* transcripts (*sgg-RA*, *sgg-RB*, *sgg-RG*, and *sgg-RO*) on *Drosophila* lifespan and aging. Different *sgg* transcripts demonstrated stage- and tissue-specificity; for example, *sgg-RA* affected lifespan only when overexpressed in muscles, and *sgg-RO* affected lifespan only when overexpressed in embryos. The sex-specific increase in adult lifespan was observed due to increased *sgg-RO* expression in embryos. Altering *sgg-RO* expression in embryos was enough to affect adult lifespan, which suggested the existence of some carry-over mechanisms of an epigenetic or other nature. The essential *sgg-RB* transcript was functional in all tissues tested, and in the nervous system, only *sgg-RB* overexpression affected lifespan, causing severe shortening. If overexpression took place in middle-aged adult flies, detrimental effects were alleviated compared to those of lifelong overexpression. Overexpression of *sgg-RB* in different types of neurons accelerated the aging of flies in a neuron-specific manner, with the most pronounced effects in dopaminergic neurons and the least pronounced effects in GABAergic neurons. Pan-neuronal *sgg-RB* overexpression severely violated the structural and functional properties of the nervous system: The integrity of neuron bodies, the number, distribution, and structure of mitochondria, cytoskeletal properties in the presynaptic zones, and synaptic activity. Overall, our data demonstrated a wide variety of effects of GSK3 on survival and proved its causal role in the aging of the nervous system.

## 2. Results and Discussion

### 2.1. Differential Expression of Sgg Affects Lifespan

According to the annotation given in FlyBase (Available online: https://flybase.org/reports/FBgn0003371), *sgg*, like many other genes, can potentially form many transcripts. Questions about what set of transcripts exist in a living organism, whether each transcript has specific functions that are different from those of other transcripts—in particular, whether the transcripts are specific for a given stage of development, age, and tissue—remain open. Our primary goal was to understand what *sgg* transcripts that are functional in the nervous system affect lifespan. However, to obtain a broader view, we tested the effects of some *sgg* transcripts in a restricted set of other tissues and at different stages. Our choice of transcripts was based on their presence in GenBank as full-size cDNAs, which indicated that these variants are actually formed when the gene is expressed. Our choice of additional tissues was based on the fact that the fat body was repeatedly shown to play an important role in lifespan control [24,25,26], whereas the role of muscles, on the contrary, is not broadly known [27]. Finally, to test the functionality of *sgg* transcripts, we used a complex phenotypic trait—lifespan.

A transgenic line providing overexpression of the essential *sgg* transcript *sgg-RB*, sggB, constructed by [19], was obtained from the Bloomington *Drosophila* Stock Center, USA (Available online: http://flystocks.bio.indiana.edu/) together with the corresponding control line (Control B). To assess the functionality of *sgg-RA*, *sgg-RG*, and *sgg-RO*, we obtained three transgenic lines providing overexpression of these transcripts: sggA, sggG, and sggO; the line initially used for transformations was used as a control line (Control AGO). We also acquired two lines with transgenic constructs providing *sgg* knockdown (sggKD1 and sggKD2), together with the control lines (Control KD1 and Control KD2) provided by the same manufacturer (Available online: http://flystocks.bio.indiana.edu/Browse/TRiPtb.htm).

Overexpression of *sgg-RA*, *sgg-RB*, *sgg-RG*, or *sgg-RO* induced by GAL4 drivers should specifically increase the amount of the corresponding transcripts, while knockdown induced by GAL4 drivers should decrease the amount of all *sgg* transcripts. The level of decrease was expected to depend on the effectiveness of the knockdown provided by different lines. sggKD1 was expected to provide a stronger level of knockdown and sggKD2 the weaker level of knockdown, according to the manufacturer’s description (https://bdsc.indiana.edu/stocks/rnai/rnai_n_z.html). A detailed description of all the lines is given in the Materials and Methods section.

To roughly characterize *sgg* effects on lifespan, we induced transcript-specific *sgg* overexpression and overall *sgg* knockdown in embryos (the D1 driver line) and in three tissues: The fat body (the D2 driver line), muscles (the D3 driver line), and the nervous system (the D4 driver line), using the GAL4-UAS binary system [28].

**Figure 1:**
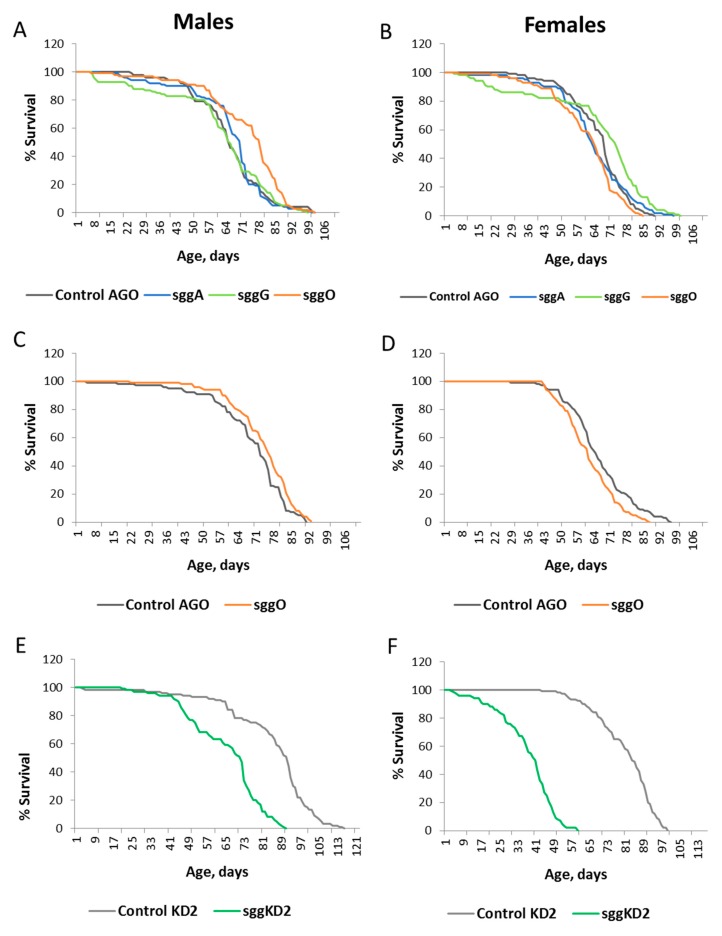
Effects of disordered *sgg* expression in embryos on male (**A**,**C**,**E**) and female (**B**,**D**,**F**) lifespans. Control AGO, sggA, sggG, sggO, Control KD2, and sggKD2 denote hybrid genotypes obtained as a result of crossing the corresponding lines with the driver line D1 inducing the expression of transgenic constructs in embryos. A full description of the genotypes is given in the Materials and Methods section.

Overexpression of *sgg-RB* in embryos was lethal, and overexpression of *sgg-RA* and *sgg-RG* had no effect on the lifespan of both males and females (Table S1, Figure 1A,B). Overexpression of *sgg-RO* affected male and female lifespans in opposite directions: In males, lifespan was significantly increased, while in females, lifespan was decreased (Table S1, Figure 1A,B). In the second experiment aimed to verify this result, the effects were reproduced, although the level of the effects was somewhat lower in both males and females (Table S1, Figure 1C,D). As expected, the effect of the overall *sgg* knockdown was lethal when the stronger sggKD1 line was used, while the weaker knockdown decreased the lifespans of both males and females (Table S1, Figure 1E,F).

**Figure 2:**
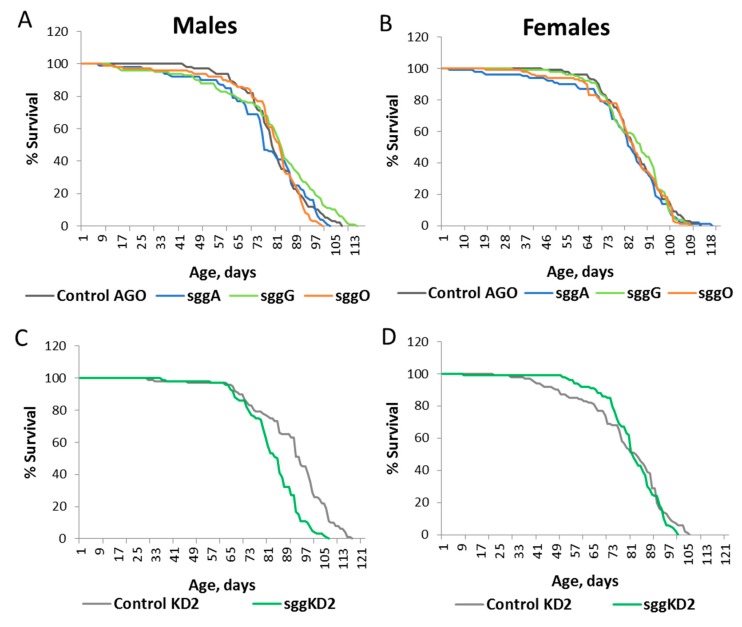
Effects of disordered *sgg* expression in the fat body on male (**A**,**C**) and female (**B**,**D**) lifespans. Control AGO, sggA, sggG, sggO, Control KD2, sggKD2, Control B, and sggA81T denote hybrid genotypes obtained as a result of crossing the corresponding lines with the driver line D2 inducing the expression of transgenic constructs in the fat body. A full description of genotypes is given in the Materials and Methods section. In panels **E** and **F**, the results of the two independent experiments are represented by solid and dotted lines.

Overexpression of *sgg-RB* in the fat body was lethal, and overexpression of *sgg-RA*, *sgg-RG*, and *sgg-RO* had no effect on the lifespans of both males and females (Table S2, Figure 2A,B). Strong *sgg* knockdown in the fat body caused lethality, while weak knockdown decreased the male lifespan and did not affect the female lifespan (Table S2, Figure 2C,D). In females, the survival curve indicated a possible positive effect of the reduced *sgg* function on lifespan.

**Figure 3:**
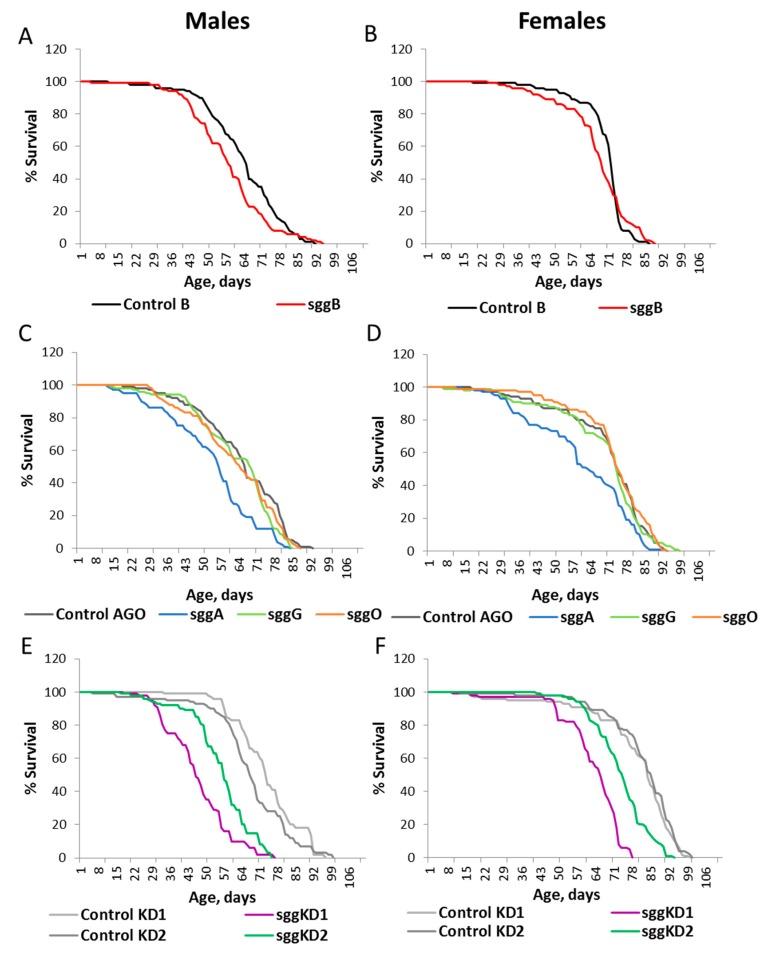
Effects of disordered *sgg* expression in muscles on male (**A**,**C**,**E**) and female (**B**,**D**,**F**) lifespans. Control B, sggB, Control AGO, sggA, sggG, sggO, Control KD1, sggKD1, Control KD2, and sggKD2 denote hybrid genotypes obtained as a result of crossing the corresponding lines with the driver line D3 inducing the expression of transgenic constructs in muscles. A full description of the genotypes is given in the Materials and Methods section.

Overexpression of *sgg-RA* and *sgg-RB* in muscles decreased both male and female lifespans, and overexpression of *sgg-RG* and *sgg-RO* had no effect on the lifespans of both males and females (Table S3, Figure 3A–D). A negative effect of *sgg-RA* overexpression was confirmed in the second experiment (Table S3). Both strong and weak knockdown in muscles decreased male and female lifespans to varying degrees (Table S3, Figure 3E,F).

**Figure 4:**
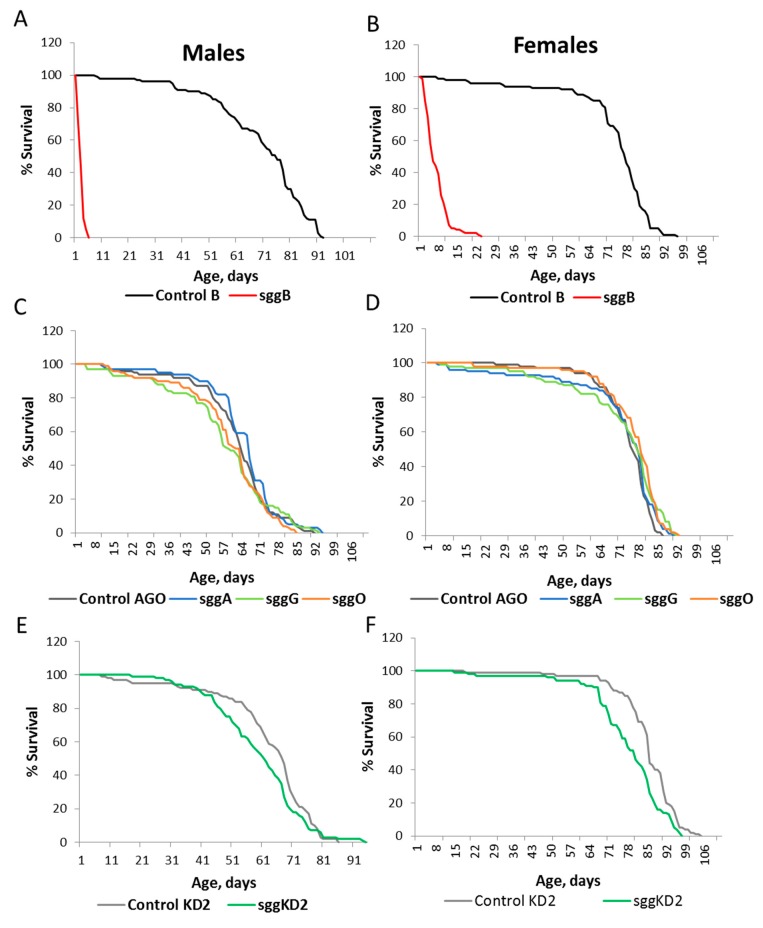
Effects of disordered *sgg* expression in the nervous system on male (**A**,**C**,**E**) and female (**B**,**D**,**F**) lifespans. Control B, sggB, Control AGO, sggA, sggG, sggO, Control KD2, and sggKD2 denote hybrid genotypes obtained as a result of crossing the corresponding lines with the driver line D4 inducing the lifelong expression of transgenic constructs in the nervous system. A full description of genotypes is given in the Materials and Methods section.

Overexpression of *sgg-RB* in the nervous system severely reduced male and female lifespans (Table S4, Figure 4A,B), and overexpression of *sgg-RA*, *sgg-RG*, and *sgg-RO* had no effect on the lifespan of both males and females (Table S4, Figure 4C,D). Strong *sgg* knockdown in the nervous system caused lethality, while weak knockdown decreased both male and female lifespans (Table S4, Figure 4E,F). Of note, weak knockdown in the nervous system affected the lifespan to a lesser degree than in muscles and in embryos, where the effect was most pronounced.

**Figure 5:**
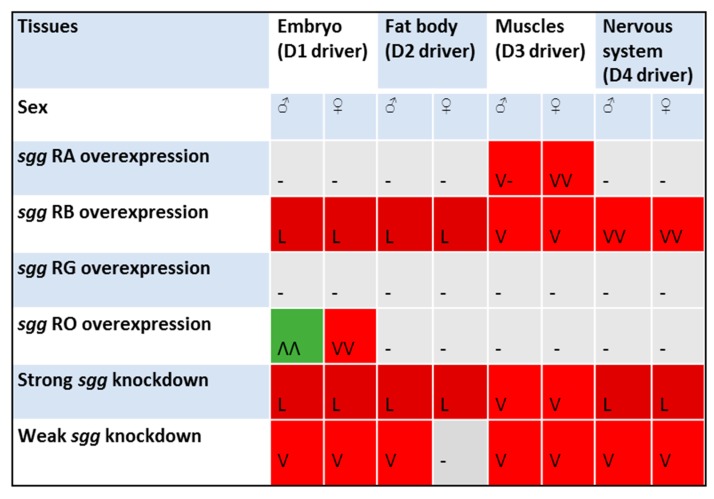
Effects of disordered *sgg* expression in different tissues on male and female lifespans. Dark-red cells, L: lethal effects; red cells, V: lifespan significantly decreased; gray cells, -: no effect; green cells, Λ: lifespan significantly increased. The number of V or Λ characters in a cell denotes the number of independent experiments.

Overall, a set of lifespan assays produced several noteworthy results (Figure 5). First, the effects of *sgg* misexpression on lifespan were mostly detrimental. The most adverse results, that is, lethality, were observed when either overexpression or knockdown were induced in embryos or in the fat body. However, increased *sgg-RO* expression in embryos increased the adult lifespan in a sex-dependent manner. Detailed research aimed at analyzing the molecular mechanisms underlying this positive effect is currently under way. The effects of gene expression early in life on lifespan are of particular interest for us because we have previously shown that changes in gene expression in embryos can lead to a sex-specific increase in longevity [16]. The sex-dependent effects of alterations in gene transcription on lifespan have often been observed by others, although the nature of this dependence remains unclear [29].

Second, different transcripts had their own patterns of effects on lifespan. This fact confirmed that *sgg* functions in a cell-autonomous mode [19]. As expected, the previously well described [7,13,18,19] *sgg-RB* transcript affected lifespan when overexpressed in all tissues and at all stages tested, although the strength of the effect varied depending on the stage, age, and tissue. In the nervous system, *sgg-RB* appeared to be the only transcript with effects on lifespan. In embryos, of all minor transcripts tested, only the expression of *sgg-RO* affected the lifespan. As its effects were not detected in other tissues, we assume that this transcript may be specific for embryos and have a function that complements *sgg-RB* functions during development. Similarly, the *sgg-RA* transcript appeared to be functionally specific for muscles, where it also may complement *sgg-RB* functions. Transcripts that are specific for or preferred at certain developmental stages and particular tissues and organs have long been known (for example, [30,31,32]). The peculiarity of our data is that they allowed us to show in which tissues these transcripts are functional, judging by the integral phenotype used in our tests. Only one transcript, *sgg-RG*, did not show functionality in our tests, which, however, does not mean that it is generally deprived of any function, given the limited scope of our tests. Of note, we were able to overexpress individual transcripts, but knockdown reduced the expression of *sgg* transcripts all together; it would be interesting to evaluate the effects of decreased expression of individual transcripts on lifespan and other traits. We have already demonstrated that reduced transcription of a gene causes lifespan extension, whereas overexpression has detrimental effects [27].

The fact that in the nervous system, only *sgg-RB* appeared to be functional gave us the necessary grounds for further analysis of the effects of *sgg* misexpression in the nervous system on the lifespan and structural and functional properties of neurons.

### 2.2. Overexpression of sgg-RB in the Nervous System Affects Lifespan in a Stage/Age- and Neuron-Specific Manner

We focused our efforts on the study of *sgg* overexpression in the nervous system because increased GSK3 function is thought to be associated with the development of several age-dependent pathological conditions, such as Alzheimer’s disease and Parkinson’s disease [12,33,34]. The increase in the number of *sgg-RB* transcripts and amount of GSK3 protein induced by the D4 panneuronal driver line was confirmed using real-time RT-qPCR and Western blotting, respectively (*p* < 0.0001 for comparisons between control and overexpressing individuals in both cases). Typical results of real-time RT-qPCR and Western blotting are illustrated in Figure S1.

**Figure 6:**
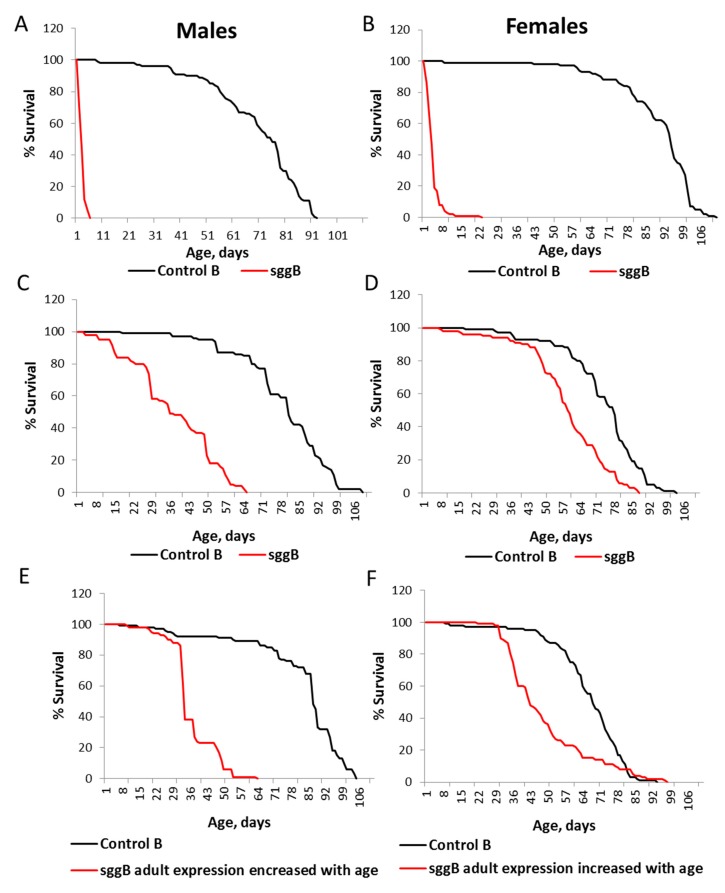
Effects of *sgg-RB* overexpression in the nervous system on male (**A**,**C**,**E**) and female (**B**,**D**,**F**) lifespans. Control B, sggB denote hybrid genotypes obtained as a result of crossing the corresponding lines with the driver line D4 inducing the lifelong expression of the transgenic construct in the nervous system (**A**,**B**), with the driver line D5 inducing the expression of the transgenic construct in the embryonic CNS (central nervous system) (**C**,**D**), and with the driver line D6 inducing the increasing expression of the transgenic construct in the adult nervous system with age (**E**,**F**). A full description of genotypes is given in the Materials and Methods section.

Severe effects of *sgg-RB* overexpression in the nervous system on male and female lifespans were confirmed in the independent experiment, which was performed approximately a year after the first experiment (Table S4, Figure 6A,B). In both replicate experiments, the effects in males were more pronounced (Figure 4A,B and Figure 6A,B); however, flies of both sexes lived only several days.

The *elav* driver (D4) used in the experiments described above provides lifelong expression in the nervous system, starting from the embryonic stage (see references at http://flybase.org/reports/FBti0002575.html).; therefore, we were not able to discriminate at which stage/age *sgg-RB* overexpression was significant for lifespan effects. To address this question, we used two additional drivers that induce the expression of transgenic constructs in the embryonic CNS (central nervous system) (D5; see references at http://flybase.org/reports/FBti0001255.html). and the increased with age expression of transgenic constructs in the adult nervous system (D6; see references at http://flybase.org/reports/FBti0040381.html). *sgg-RB* overexpression in the embryonic CNS decreased male and female lifespans, though to a lesser degree than lifelong panneuronal overexpression (Table S4, Figure 6C,D). This result indicated that normal *sgg-RB* expression in the CNS at the embryonic stage is important for lifespan determination; however, it does not fully account for the effects of lifelong panneuronal *sgg-RB* overexpression. According to the description given in FlyBase, the D6 driver should be switched on starting from the 50th day of life. However, survival curves obtained in our experiments (Table S4, Figure 6E,F) indicated that both control flies and flies with the *sgg-RB* transgene lived the same way until approximately the 30th day of life, but after that, the lifespans of both males and females with the *sgg-RB* transgene declined significantly faster compared to the control. These data clearly indicate that the D6 driver was switched on starting from the 30th day of life and that *sgg-RB* overexpression starting in the nervous system of middle-aged flies was sufficient to decrease their lifespans, though to a lesser degree than lifelong overexpression: The slope of survival curves in the latter case was much steeper both in males and females.

Overall, *sgg-RB* overexpression in the nervous system at different stages of development and aging negatively affected lifespan. Altering *sgg-RB* expression in the embryonic CNS was enough to affect the adult lifespan, which could be explained by epigenetic inheritance in cell lines or other yet unknown mechanisms. Based on our results, it is tempting to conclude that in the nervous system, embryonic *sgg-RB* overexpression is less detrimental than lifelong *sgg-RB* overexpression and *sgg-RB* overexpression in adults and that if overexpression took place in older adults, detrimental effects were alleviated. One of the theories of aging suggests that aging is caused by hyperfunction, that is, overactivity during adulthood of processes that are primarily important during development. Such hyperfunction can lead to hypertrophy-associated pathologies, which cause accelerated aging [35]. In the most general terms, smaller effects of overexpression in embryos would be in accordance with this theory. However, the strength of overexpression induced by different drivers in our experiments may be different and bias the comparisons. Experiments with a conditional tissue-specific transgene expression system using the elav-geneswitch driver could allow overexpression in larvae and adults at different ages and provide adequate comparison of the stage- and age-specific effects of altered *sgg-RB* expression. To compare the effects of overexpression in embryos and in adult flies, more sophisticated methods are needed, for example, combinations of GAL4 and GAL80 or split GAL4 drivers with different stage-specific patterns of expression, in accordance with the logic proposed in [36]. One cannot exclude the possibility that *sgg-RB* overexpression in old flies could become beneficial. Future experiments can shed light on these issues.

The *elav* driver (D4) provides panneuronal expression (see references at http://flybase.org/reports/FBti0002575.html); therefore, we were not able to discriminate which neurons are most sensitive to *sgg-RB* overexpression and are predominantly responsible for effects on lifespan. To address this question, we used several drivers (D7–D13) that induce the expression of transgenic constructs in neurons secreting different transmitters and in motor neurons. Two different drivers were used to induce *sgg-RB* overexpression in dopaminergic neurons, and in both cases, the effect was lethal. These neurons demonstrated maximum sensitivity to the level of *sgg-RB* expression. It remained unclear why in this case the panneuronal *sgg-RB* overexpression was not lethal; one mechanistic suggestion would be that in some neurons, *sgg-RB* overexpression had a positive effect on lifespan and that opposite effects counterbalanced each other. However, *sgg-RB* overexpression in all tested types of neurons decreased both male and female lifespans to varying degrees (Table S5, Figure S2). Overall, peptidergic neurons appeared to be highly sensitive; glutamate and cholinergic neurons, less sensitive; and motor and GABAergic neurons, the least sensitive to *sgg-RB* overexpression among the types of neurons tested, judging by the effect on lifespan. However, the level of the effect also depended on sex (Figure S2). Other types of differently classified neurons might be worth testing in the future to better understand the specific impact of neurons on lifespan control provided by Sgg-RB.

Our results demonstrated that, despite the individual nuances, lifelong panneuronal *sgg-RB* overexpression would be an appropriate model system for further analyses of GSK impact on structural and functional properties of neurons. To better understand the association between the extent of lifespan reduction and alterations in neuronal characteristics, for further analyses, we also selected overexpression of *sgg-RB* in motor neurons as a second model system demonstrating moderate effects on lifespan. The choice of this additional model was also justified by the fact that we used larval NMJs (neuromuscular junctions) as a model synapse.

### 2.3. Overexpression of Sgg-RB in the Nervous System Affects Neuronal Structure and Function

We suggest that the lifespan reduction caused by aberrations in GSK3 expression in the nervous system is based on pathological changes in neurons. It was previously shown that *sgg* overexpression results in decreased numbers of synaptic boutons [7] and synapses [9], small synapses [8], and degeneration of presynaptic terminals and axons [10]. We decided to complement these results by some other structural and functional characteristics of the nervous system in flies with a dramatically decreased lifespan due to *sgg-RB* panneuronal overexpression and in flies with a moderately decreased lifespan due to *sgg-RB* overexpression in motor neurons.

#### 2.3.1. Locomotion

**Figure 7:**
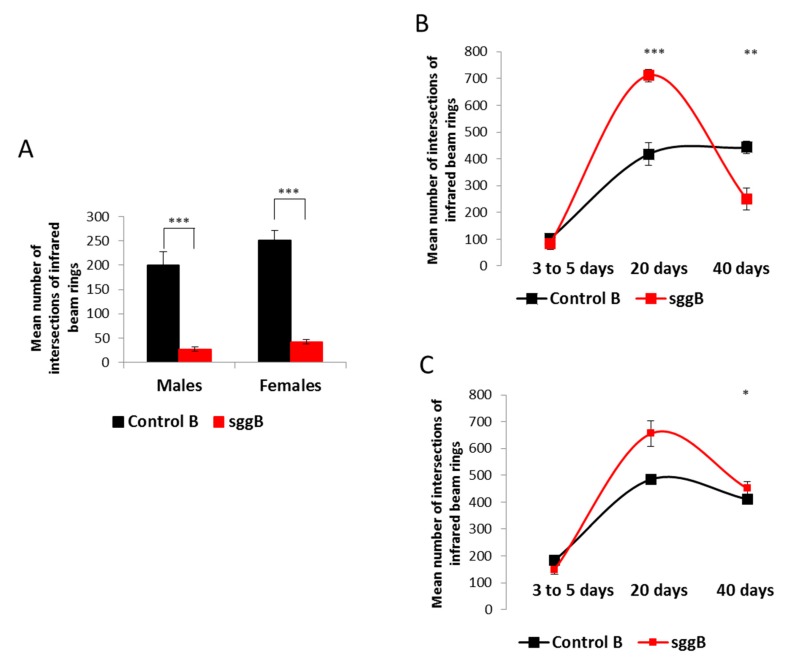
Effects of *sgg-RB* overexpression in the nervous system on locomotion of flies. Panneuronal *sgg-RB* overexpression in 1- to 3-day-old males and females (**A**). *sgg-RB* overexpression in motor neurons in males (**B**) and females (**C**). Control B, sggB denote hybrid genotypes obtained as a result of crossing the corresponding lines with the driver line D4 inducing the expression of transgenic constructs in the nervous system or the driver line D12 inducing the expression of transgenic constructs in motor neurons. A full description of genotypes is given in the Materials and Methods section. * denotes *p* < 0.05, ** denotes *p* < 0.01, *** denotes *p* < 0.001 determined by the Kruskal-Wallis test.

First, we demonstrated that locomotion was highly impaired in 3- to 5-day-old males and females with panneuronal overexpression of *sgg-RB* (Figure 7A), indicating severe lesions in the functional status of the nervous system. These flies did not live longer than 10 days (males) or 15 days (females), and most of them hardly moved at all in the second half of life, so we were not able to characterize the age-dependent dynamics of their locomotion. In 3- to 5-day-old males and females with *sgg-RB* overexpression in motor neurons, locomotion was not changed compared to that of control flies, and in 20-day-old flies, locomotion was significantly increased compared to that of control flies (Figure 7B,C). In 40-day-old control flies, locomotion was still high, while in flies with *sgg-RB* overexpression in motor neurons, a substantial decline in locomotion was observed (Figure 7B,C). Of note, the mean lifespan of control males and females was 64 ± 3 and 68 ± 2 days, respectively (Table S4), and at the age of 40 days, the flies were not truly aging. The mean lifespan of males with *sgg-RB* overexpression in motor neurons was 35 ± 2 days (Table S4), and 40-day-old males were intensively aging, which was reflected in the rapid decline in locomotion that decreased below the control level. The mean lifespan of females with *sgg-RB* overexpression in motor neurons was 58 ± 2 days (Table S4), and the biological age of 40-day-old females was younger than in males, which was reflected in a smaller decline in locomotion that stayed at the control level. Overall, in our experiments, locomotion, as reported by [37], appeared to be a good marker of aging.

At the same time, an intriguing result of these experiments was that in 20-day-old flies with *sgg-RB* overexpression in motor neurons, the presumed decline in neuronal functions [7,8,9,10] and decreased lifespan were associated with elevated locomotion, indicating that the functional status of the nervous system necessary for ensuring motor functions was not only disturbed, but even improved. Simultaneous decreases in lifespan and increases in locomotion, both resulting from changes in the expression of a gene, have been reported previously [37]. It was suggested that mitochondrial function in neurons may change in such a way that the lifespan decreases due to the increased ROS (reactive oxygen species) production known to promote aging and shorten lifespan [38,39], whereas locomotion increases due to elevated mitochondrial activity and ATP levels. Indeed, the increase in mitochondrial function to a certain level should result in the increase of energy production accompanied by elevated levels of ROS. However, elevated ROS and ATP levels did not accompany a decrease in lifespan and an increase in locomotion described in [37], and the cellular mechanisms underlying an increase in locomotion remained obscure.

#### 2.3.2. Mitochondria

**Figure 8:**
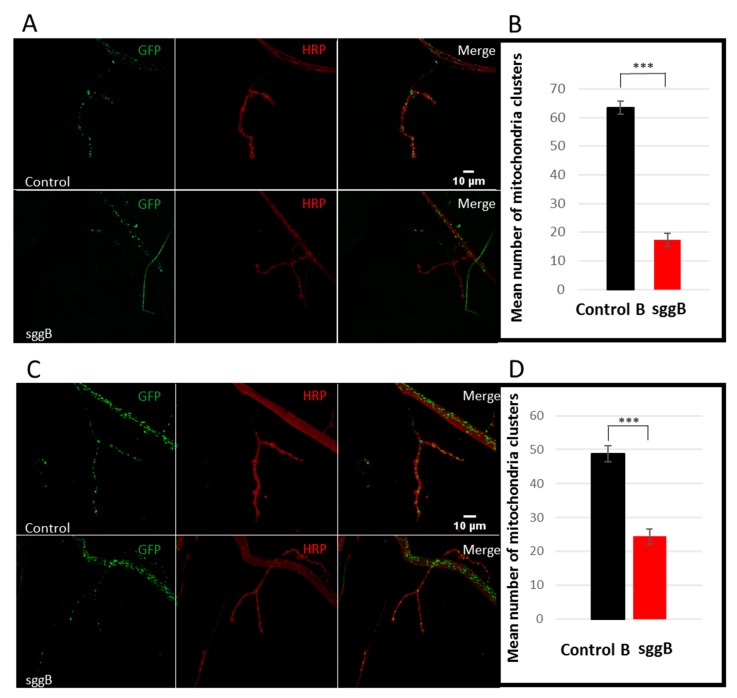
Mitochondrial clusters in NMJs of third-instar female larvae with panneuronal *sgg-RB* overexpression (**A**,**B**) and *sgg-RB* overexpression in motor neurons (**C**,**D**). Control B, sggB denote hybrid genotypes obtained as a result of crossing the corresponding lines with the driver line D4 inducing the expression of transgenic constructs in the nervous system or the driver line D12 inducing the expression of transgenic constructs in motor neurons. A full description of genotypes is given in the Materials and Methods section. Representative confocal images of NMJs (muscle 4, hemi-segment 34–) stained for mitochondria clusters (GFP, green) and neural membranes (HRP, horseradish peroxidase, red) (**A**,**C**). Bar = 10 µm. Quantification of the number of mitochondrial clusters (**B**,**D**). *** denotes *p* < 0.001 determined by the Kruskal-Wallis test.

**Figure 9:**
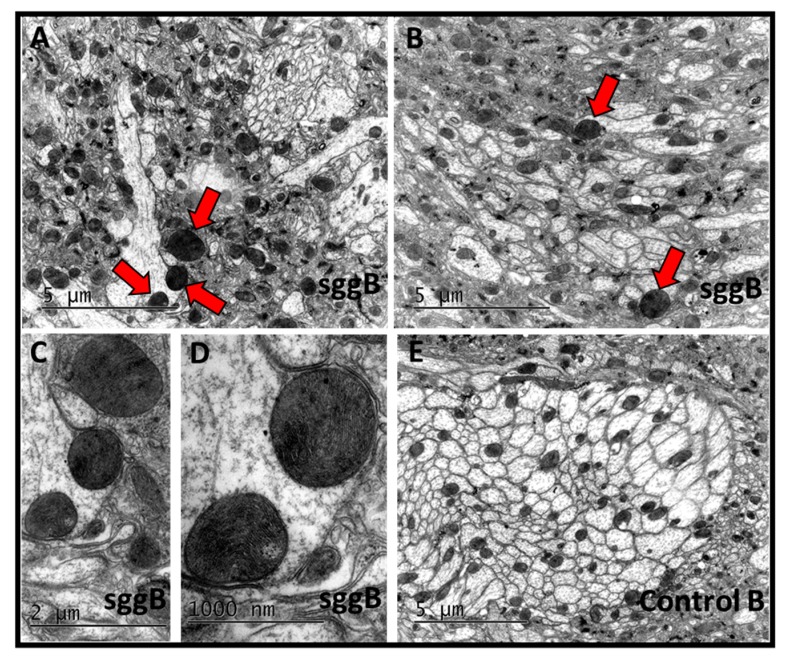
Fine structure of the adult brain in females with pan-neuronal *sgg-RB* overexpression. Control B, sggB denote hybrid genotypes obtained as a result of crossing the corresponding lines with the driver line D4 inducing the expression of transgenic constructs in the nervous system. A full description of genotypes is given in the Materials and Methods section. Three representative transmission electron microscopy images of the *Drosophila* inferior dorsofrontal protocerebrum at the age of 3 days (**A**,**B**,**E**); enlarged fragments of panel B (**C**,**D**). Mitochondria with violated structures are indicated by red arrows. Bar = 5 µm or 2 µm.

The functionality of neurons critically depends on the energy supply, which is ensured by the work of mitochondria. Mitochondrial homeostasis, that is, precise control of mitochondrial number, integrity, and distribution, is especially critical to postmitotic cells, such as neurons. Violation of the fine-tuned interplay between mitochondrial function, energy metabolism, and neuronal activity is critical in the occurrence of various neurological pathologies [40]. We examined whether the number of mitochondria was affected by *sgg-RB* overexpression. For this purpose, we used larval NMJs, which are often used as a model system to study synapse development and function in *Drosophila* [41]. GSK3 is enriched in the presynaptic side of NMJs [7], so we focused on studying the presynaptic zone. Mitochondria are especially abundant at the presynaptic parts of axons. The number of GFP-labeled mitochondrial clusters was decreased in NMJs of individuals with panneuronal *sgg-RB* overexpression (Figure 8A,B) and *sgg-RB* overexpression in motor neurons (Figure 8C,D) to a similar extent. Thus, the decreased number of mitochondrial clusters was associated with both severe and moderate decreases in lifespan, but appeared to be not relevant to locomotion. Analysis of the brains of 3-day-old flies revealed swollen, dense mitochondria in all three individuals with pan-neuronal *sgg-RB* overexpression (Figure 9A–D). We failed to observe such mitochondria in control individuals (Figure 9E).

In individuals with *sgg-RB* overexpression, mitochondria were clustered predominantly in synaptic boutons, possibly to compensate for the overall decrease in number, whereas in control individuals, they were distributed more evenly along terminal ends of axons (Figure 8A,C). Mitochondria are produced in the neuronal soma and move to synapses, where they are needed for neuronal firing, along microtubules using kinesin motors; they can also move in the retrograde direction for reparation using dynein motors [42]. GSK3β is involved in the control of mitochondrial movement, both in increasing upon activation of the serotonin receptor [43] and in decreasing upon activation of the dopamine receptor [44,45]. GSK3β directly regulates dynein [46] and promotes anterograde movement [47]. We hypothesize that *sgg-RB* overexpression might affect mitochondrial movement and thus be responsible for disturbances in the distribution of mitochondria along axons.

#### 2.3.3. Cytoskeleton

**Figure 10:**
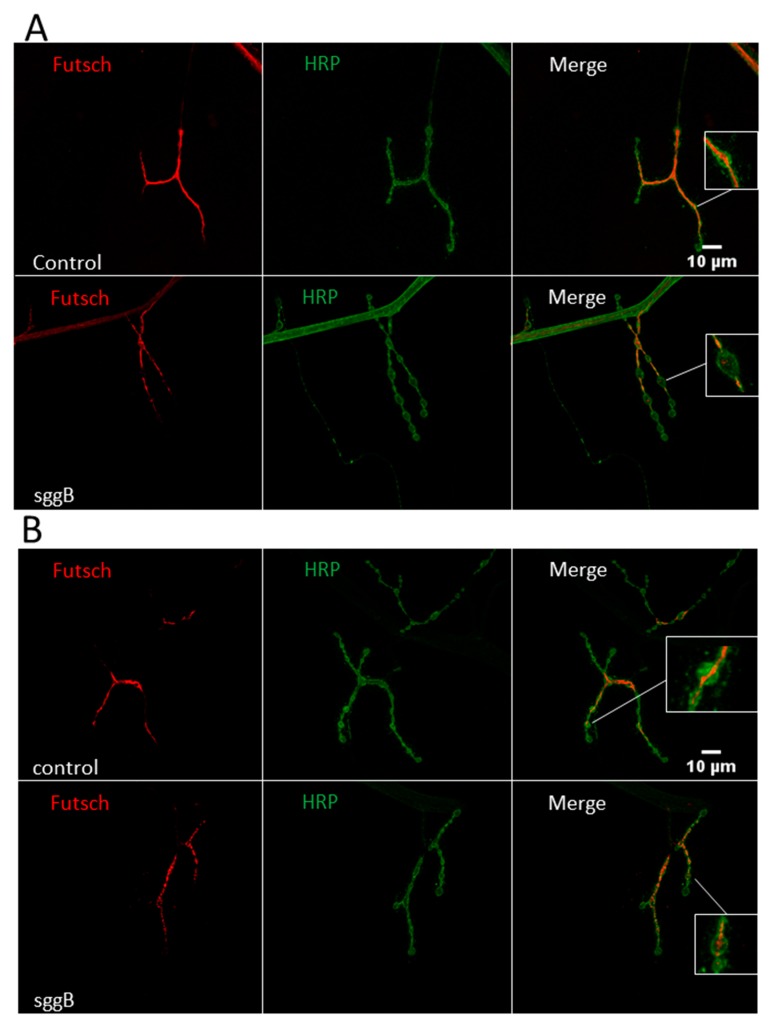
Distribution of Futsch in NMJs of third-instar female larvae with panneuronal *sgg-RB* overexpression (**A**) and *sgg-RB* overexpression in motor neurons (**B**). Control B, sggB denote hybrid genotypes obtained as a result of crossing the corresponding lines with the driver line D4 inducing the expression of transgenic constructs in the nervous system or the driver line D12 inducing the expression of transgenic constructs in motor neurons. A full description of genotypes is given in the Materials and Methods section. Representative confocal images of NMJs (muscle 4, hemi-segment 34–) stained for Futsch (red) and neural membranes (HRP, green). Bar = 10 µm.

Not only mitochondrial movement, but also the overall functionality and architecture of neurons depend on the integrity of their cytoskeletons. We assessed the effects of *sgg-RB* overexpression in individuals with pan-neuronal *sgg-RB* overexpression and *sgg-RB* overexpression in motor neurons on the morphology of the microtubule network in NMJs, as visualized by antibodies to α-acetylated tubulin and to Futsch, a neuronal microtubule-associated protein [48]. Staining with antibodies to α-acetylated tubulin failed to reveal visual differences between the individuals with and without *sgg-RB* overexpression (Figure S3). Differences in the distribution of Futsch within the NMJs were found. In particular, in individuals with *sgg-RB* overexpression, only weak, diffuse Futsch staining in synaptic boutons of NMJs was observed compared to that in controls (Figure 10), indicating cytoskeletal abnormalities. In this case, the effect was more pronounced in individuals with pan-neuronal *sgg-RB* overexpression (Figure 10A) than in individuals with *sgg-RB* overexpression in motor neurons (Figure 10B), which correlates with the comparative effect on lifespan. It was shown that *sgg* interacts with Futsch, which functions downstream of *sgg* [7]; most likely, *sgg* controls synaptic growth through the direct phosphorylation of Futsch. Sgg is able to inhibit transcription factor AP-1 and the JNK (Jun-N-terminal kinase) cascade and, in this way, also affects synaptic growth [8]. Our data shed light on the cellular consequences of presumed increased phosphorylation of Futsch, which disappeared from synaptic boutons of NMJs, and demonstrated that Sgg-RB, when overexpressed, controlled the dynamics of the microtubule cytoskeleton.

#### 2.3.4. Active Synaptic Zones

**Figure 11:**
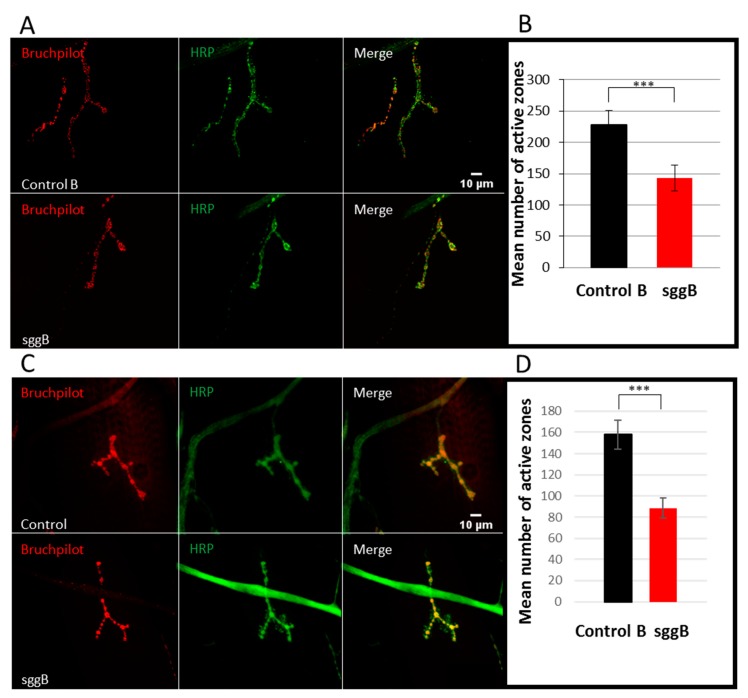
Active zones in NMJs of third-instar female larvae with panneuronal *sgg-RB* overexpression (**A**,**B**) and *sgg-RB* overexpression in motor neurons (**C**,**D**). Control B, sggB denote hybrid genotypes obtained as a result of crossing the corresponding lines with the driver line D4 inducing the expression of transgenic constructs in the nervous system or the driver line D12 inducing the expression of transgenic constructs in motor neurons. A full description of genotypes is given in the Materials and Methods section. Representative confocal images of NMJs (muscle 4, hemi-segment 34–) stained for active zones (BRP, red) and neural membranes (HRP, green) (**A**,**C**). Bar = 10 µm. Quantification of the number of active zones (**B**,**D**). *** denotes *p* < 0.001 determined by the Kruskal-Wallis test.

Given that the number and size of synapses were shown to be affected by *sgg* function [7,8], we decided to evaluate the number of active synaptic zones in individuals with *sgg-RB* overexpression. The number of active zones in larval NMJs visualized by antibodies to BRP (Bruchpilot), a Drosophila homolog of vertebrate active zone protein ELKS [29], was significantly (approximately two-fold) decreased in individuals with pan-neuronal *sgg-RB* overexpression (Figure 11A,B) and *sgg-RB* overexpression in motor neurons (Figure 11C,D), indicating reduced synaptic activity. *sgg-RB* overexpression driven by both drivers had comparable effects on NMJs. These results supplemented the data on the role of Sgg in controlling the functionality of synapses, which demonstrated that it was involved in the regulation of neurotransmitter release [8]. Earlier, it was shown that the number of boutons was significantly decreased as a result of pan-neuronal GSK3 overexpression [7]. Importantly, although *sgg* overexpression has an inhibitory role on the quantity of boutons, it may not play an important role in bouton differentiation [7]. Overall, our data demonstrated that negative changes in lifespan caused by Sgg-RB overexpression in the nervous system were accompanied by negative changes in synaptic properties.

#### 2.3.5. Neuronal Structure

**Figure 12:**
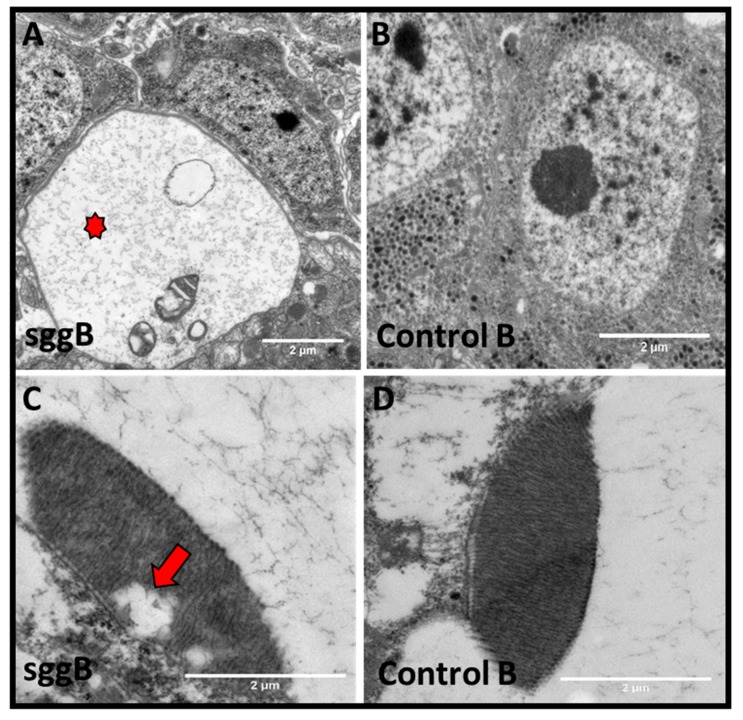
Signs of degeneration in the nervous system of females with pan-neuronal *sgg-RB* overexpression. Control B, sggB denote hybrid genotypes obtained as a result of crossing the corresponding lines with the driver line D4 inducing the expression of transgenic constructs in the nervous system. A full description of genotypes is given in the Materials and Methods section. Representative transmission electron microscopy images of the *Drosophila* brain (**A**,**B**) and rhabdomeres (**C**,**D**) at the age of 3 days. An example of a degeneration sign in the neuron body (**A**, indicated by the red star); rhabdomeres with violated structure (**C**, lacuna is indicated by the red arrow) compared to the respective controls (**B**,**D**). Bar = 1 µm or 2 µm.

We suggest that a significant decrease in the lifespan of individuals with pan-neuronal *sgg-RB* overexpression might be accompanied by pathological processes in the adult nervous system. Analysis of the brains of 3-day-old flies using transmission electron microscopy revealed a number of degenerative marks both in the bodies of the nerve cells and in the axonal structures in the neuropil region. The presence of “holes” surrounded by membranes and, in some cases, containing fragments of organelles apparently subjected to autophagy was demonstrated in the brains of all three individuals with pan-neuronal *sgg-RB* overexpression, whereas these structures were not found in the control brains (Figure 12A,B). These “holes” apparently represent destroyed areas of the neuron bodies and may indicate accelerated aging processes in the nervous system of individuals with pan-neuronal *sgg-RB* overexpression. In *Drosophila*, photosensitive structures, namely, rhabdomeres, are often used along with the brain to study neurodegenerative processes. We examined whether pathological changes occur in the rhabdomeres of individuals with pan-neuronal *sgg-RB* overexpression compared to those of controls. No changes were revealed in three control individuals, whereas in two out of three individuals with pan-neuronal *sgg-RB* overexpression, lacunae and irregular rhabdomere structures were found (Figure 12C,D), also indicating pathological processes in flies with pan-neuronal *sgg-RB* overexpression. Of note, in individuals with pan-neuronal *sgg-RB* overexpression, a high heterogeneity of external phenotypic manifestations was observed: For example, the wing shape varied fairly widely. In view of this fact, some difference in the level of degeneration of rhabdomeres was not surprising.

Summing up the results presented in this chapter, we conclude that overexpression of the main Sgg-RB isoform in the nervous system had severe effects on its structural and functional properties. Overall, Sgg-RB overexpression in the nervous system affected the integrity of neuron bodies, the number, distribution, and structure of mitochondrial and cytoskeletal properties in the presynaptic zones, and synaptic activity. These changes indicated that Sgg-RB overexpression caused accelerated aging of the nervous system in young individuals. The accelerated aging led to a more or less sharp reduction in lifespan, depending on the cell types where Sgg-RB overexpression was induced. While pan-neuronal overexpression caused a severe lifespan decline and overexpression in motor neurons reduced the lifespan to a much lesser extent, and the effects of pan-neuronal overexpression and overexpression in motor neurons on the nervous system were mostly of similar scales. Thus, the rate of lifespan decline was not correlated with the rate of structural and functional changes in the nervous system. The only exception identified was a change in the structure of the cytoskeleton, namely, the Futsch protein distribution along the terminal parts of axons.

## 3. Materials and Methods

### 3.1. Fly Strains and Crosses

To provide *sgg* overexpression, several lines were used. The line providing *sgg-RB* overexpression *w^1118^; P{w^+mC^ = UAS-sgg. B}MB5* (in short, sggB) with the transgenic construct encoding the normal PB form of GSK3 was obtained from the Bloomington *Drosophila* Stock Center (USA) (Available online: http://flystocks.bio.indiana.edu/); the *w^1118^* (Control B) line without transgenic insertions was used as a control line [19].

To obtain lines providing *sgg-RA*, *sgg-RG*, and *sgg-RO* overexpression, cDNAs corresponding to these transcripts were cloned into the *pBID-UASC* vector (Addgene plasmid # 35200, Available online: https://www.addgene.org/35200/), which contains an attB site for *phi31* site-specific transformation of *Drosophila* embryos and UAS enhancer sequences [49]. Single clones with the unimpaired *sgg-RA*, *sgg-RG* and *sgg-RO* sequences and the line y^1^ M{vas-int.Dm}ZH-2A w*; M{3xP3-RFP.attP’}ZH-51C with the second chromosome attP *phi31* integration site [50] were used for the transformation performed by BestGene, Inc. (Available online: https://www.thebestgene.com/HomePage.do). In the transgenic lines obtained, most of the auxiliary sequences were excised from the inserted transgenes due to induction of recombination between the *loxP* sites present in the *pBID-UASC* vector. One homozygous transgenic line of each genotype, namely, y^1^ M{vas-int.Dm}ZH-2A w*; M{3xP3-RFP.attP’}ZH-51C P{w^+mC^ = UAS-sgg.A}2M (in short, sggA), y^1^ M{vas-int.Dm}ZH-2A w*; M{3xP3-RFP.attP’}ZH-51C P{w^+mC^ = UAS-sgg.G}1M (in short, sggG), y^1^ M{vas-int.Dm}ZH-2A w*; and *M{3xP3-RFP.attP’}ZH-51C P{w^+mC^ = UAS-sgg.O}1M* (in short, sggO), were used in further experiments. The line y^1^ M{vas-int.Dm}ZH-2A w*; M{3xP3-RFP.attP’}ZH-51C (Control AGO) initially used for transformations was used as a control line with the same genetic background.

To provide *sgg* RNAi knockdown, two lines were obtained from the Bloomington *Drosophila* Stock Center: y^1^ sc* v^1^; P{y^+t7.7^ v^+t1.8^ = TriP. HMS01751}attP40 (in short, sggKD1, with VALIUM20, hairpin size 21 bp, affects all *sgg* transcripts) and *y^1^ v^1^; P{y^+t7.7^ v^+t1.8^ = TriP. JF01255}attP2* (in short, sggKD2, with VALIUM1, hairpin size 400 bp, affects all *sgg* transcripts). *Y^1^ v^1^; P{*y^+t7.7^ = *CaryP}attP40* (Control KD1) and *y^1^ v^1^; P{y^+t7.7^=CaryP}attP2* (Control KD2) lines without transgenes providing RNAi were used as control lines for *sgg* RNAi knockdown, as suggested by the manufacturer (Available online: http://flystocks.bio.indiana.edu/Browse/TRiPtb.htm).

To induce *sgg* overexpression or knockdown, several driver lines were obtained from the Bloomington *Drosophila* Stock Center.

Y^1^ w*; P{w^+mW.hs^ = en2.4-GAL4}e22c; P{w^+mC^ = tGPH}4/TM3, Ser^1^ (D1) was used to induce the expression of transgenic constructs in embryos.

W*; P{w^+mC^ = ppl-GAL4. P}2 (D2) was used to induce the expression of transgenic constructs in the fat body.

*P{w^+mC^ = UAS-Dcr-2. D}1, w^1118^; P{w^+mC^ = GAL4-Mef2. R}R1* (D3) was used to induce the expression of transgenic constructs in somatic muscle cells.

*P{w^+mW.hs^ = GawB}elavC155 w^1118^; P{w^+mC^ = UAS-Dcr-2. D}2* (D4) was used to induce the lifelong expression of transgenic constructs in the nervous system.

W*; P{w^+mW.hs^ = GawB}389 (D5) was used to induce the expression of transgenic constructs in the embryonic nervous system.

*W^1118^; P{GawB}DJ695* (D6) was used to induce the increased expression of transgenic constructs in the adult nervous system with age.

*W^1118^; P{w^+mC^ = Ddc-GAL4.L}Lmpt^4.36^* (D7) and w*; P{w[+mC] = UAS-mCD8::GFP. L}LL5/Cy; and *P{w^+mC^ = ple-GAL4. F}3* (D8) were used to induce the expression of transgenic constructs in dopaminergic neurons.

W*; P{w^+mW.hs^ = GawB}386Y (D9) was used to induce the expression of transgenic constructs in peptidergic neurons.

*W^1118^; P{w^+mW.hs^ = GawB}Vglut^OK371^* (D10) was used to induce the expression of transgenic constructs in glutamatergic neurons.

W*; P{w^+mC^ = ChAT-GAL4.7.4}19B (D11) was used to induce the expression of transgenic constructs in cholinergic neurons.

W*; P{GawB}D42 (D12) was used to induce the expression of transgenic constructs in motoneurons.

*P{w^+mC^ = Gad1-GAL4.3.098}2/CyO* (D13) was used to induce the expression of transgenic constructs in GABAergic neurons.

Additionally, the driver line *w^1118^;P{w^+mC^ = UAS-mito- HA-GFP.AP}2/CyO* (D14) was used to induce GFP expression in mitochondria.

D1, D3, and D4 driver lines proved to be effective in our previous work, according to the real-time RT-qPCR data [27].

To induce the expression of transgenic constructs, females of each of the driver lines were crossed to males of sggB, sggY214F, sggA81T, sggA, sggG, sggO, KD1, KD2, Control B, Control AGO, Control KD1, and Control KD2 lines. In all experiments, flies were kept at 25 °C on a standard medium of semolina, sugar, raisins, yeast, and agar with nipagin, propionic acid, and streptomycin.

### 3.2. Tests for Wolbachia

Prior to the experiments, all the lines were checked for the presence of *Wolbachia*, a *Drosophila* symbiont known to affect life history traits [51], via quantitative PCR (MiniOpticon real-time PCR detection system, Bio-Rad, Hercules, CA, USA) with primers for the 16S rRNA gene, 5′-CATACCTATTCGAAGGGATAG-3′ and 5′-AGCTTCGAGTGAAACCAATTC-3′ [52]. Negative results were obtained for all lines except sggA, D3, and D12. These lines were treated with tetracycline (0.25 mg/mL, [53]) for three generations followed by at least three generations of recovery before they were used in experiments.

### 3.3. Lifespan Assay

Lifespan was measured as described in [16]. Five virgin flies of the same genotype and sex, all collected on the same day from cultures with moderate density, were placed in replicate vials. Flies were transferred to vials with fresh food containing approximately 5 mL of standard medium without live yeast on the surface weekly. The number of dead flies was recorded daily. Experiments comparing fly lifespans were conducted simultaneously. Sample sizes were 50 to 100 flies per sex per genotype. The experiments that showed noteworthy results were repeated two to three times with an interval of approximately six months. The lifespan for each fly was estimated as the number of days alive from the day of eclosion to the day of death. Mean lifespans and survival curves were primarily used to characterize lifespan.

### 3.4. Locomotion Assay

Locomotion was measured as described in [16]. Flies were collected and maintained by the same procedures as for the lifespan assays, but without recording the deaths. Locomotion was measured at the same time each day in unmated males and females at age 3 to 5 days, 20 days and 40 days. Experiments comparing locomotion were conducted simultaneously. Sample sizes were 65 to 75 flies (13 to 15 vials) per sex per genotype. To measure locomotor activity, the vials were placed horizontally in a *Drosophila* Population Monitor (TriKinetics Inc, Waltham, MA, USA). Fly movement along the walls or in the middle of the vial crossed the infrared beam rings along the length of the vial. Beam interruptions were detected, and totals were reported every five minutes to the host computer. Two measurements for five minutes were made for each vial. Locomotion was characterized as the mean number of beam interruptions per vial.

### 3.5. Real-Time RT-qPCR

Transcript levels were measured as described in [15,16]. Total RNA for real-time reverse transcription quantitative PCR (RT-qPCR) was extracted from batches of 30 whole bodies of 3- to 5-day-old males and females using TRIzol reagent (Invitrogen, Carlsbad, CA, USA) and DNase I (Sigma-Aldrich, St. Louis, MO, USA) according to the manufacturers’ instructions.

First-strand cDNA was synthesized using SuperScript II Reverse Transcriptase (Invitrogen) with oligo(dT)_15_ primers according to the manufacturer’s instructions. Amounts of cDNA were determined by RT-qPCR using SYBR Green I in a MiniOpticon real-time PCR detection system (Bio-Rad).

*Gdh* and *Adh* housekeeping genes, characterized by relatively low expression comparable to *sgg* expression, were used as reference genes to normalize for differences in total cDNA between the samples. The forward and reverse primer sequences used were as follows: For *sggB*, shaggyPB1 5′-ATATACAGATCTTTTGTTTGGCAA-3′ and shaggyPB2 5′-AGGAGGAAGTTCTTGGACGA-3′; for *Gdh*, Gdh1 5′-TATGCCACCGAGCACCAGATTCC-3′ and Gdh2 5′-GGATGCCCTTCACCTTCTGCTTCTT-3′; for *Adh*, Adhd3: 5′-CGGCATCTAAGAAGTGATACTCCCAAAA-3′ and Adhr3: 5′-TGAGTGTGCATCGAATCAGCCTTATT-3′.

CFX Manager 3.1 software (Bio-Rad, 2012) was used to evaluate the relative gene expression. Inter-run calibrations were used for each panel of experiments since the experiments were conducted for several years. Three independent RNA extractions (biological replicates) per sex per genotype and three technical repeats for each RNA extraction were made.

### 3.6. Immunostaining and Microscopy

Immunostaining and microscopy were performed according to [27]. Male and female third-stage larvae and brains of 3- to 5-day-old unmated males and females were dissected in phosphate-buffered saline (PBS), fixed in 4% paraformaldehyde (Sigma-Aldrich, St. Louis, MO, USA) at room temperature for 20 min, and washed in PBS (3 × 15 min). For immunostaining, preparations were blocked in blocking buffer (BlockPRO, Visual Protein Biotechnology Corporation, Taiwan) for one hour at room temperature, incubated in primary antibodies (diluted in BlockPRO) overnight at 4 °C, washed in PBS (3 × 15 min), incubated in secondary antibodies (diluted in BlockPRO) for two hours, washed in PBS (3 × 15 min), and placed in a medium for immunofluorescence (VectaShield, Vector Labs, Burlingame, CA, USA). NMJs were analyzed in the fourth muscle of the third and fourth abdominal segments of larvae. A confocal laser scanning microscope (LSM 510, Zeiss, Oberkochen, Germany), ImageJ (http://rsb.info.nih.gov/ij/index.html) and LSM Image Browser (Zeiss) were used. Sample sizes were 10 to 15 specimens per genotype per experiment. To estimate bouton numbers and numbers of synaptic active zones, the same preparations were used. Mean numbers of type 1b boutons and satellite boutons were used to characterize NMJ morphology. The mean number of synaptic active zones was used to characterize synapse activity. Mean numbers of mitochondria and dopaminergic neurons were calculated.

The following primary antibodies were used: Mouse anti-Brp (mAb NC82, 1:200; Developmental Studies Hybridoma Bank (DSHB)) against Bruchpilot (BRP), a protein specific to active synaptic zones [54]; mouse anti-Futsch (mAb 22C10, 1:200; DSHB) against the microtubule-associated protein, Futsch [48]; mouse anti-α-acetylated tubulin (1:200; Santa Cruz Biotechnology, Dallas, TX, USA) against a tubulin isoform, a marker of microtubule networks [55]; Alexa Fluor 647-conjugated goat anti-HRP (1:400, Jackson ImmunoResearch), against Horseradish Peroxidase (HRP), a widely used marker of presynaptic membranes [7]. The secondary antibodies used were goat anti-mouse Cy3 conjugated (1:400, Jackson ImmunoResearch). Antibodies obtained from the DSHB were developed under the auspices of the NICHD and maintained by The University of Iowa, Department of Biology, Iowa City, IA 52242. Mitochondria were visualized with GFP using the D13 driver line. Dopaminergic neuron neurons were visualized with GFP using the D8 driver line.

### 3.7. Electron Microscopy

Three heads of 3- to 5-day-old unmated males and females of each genotype were dissected and fixed in 2.5% glutaraldehyde in cacodylate buffer at room temperature for 2 h. Samples were washed in sodium cacodylate buffer, fixed in 2% OsO_4_ for 2 h at 4 °C, washed and, dehydrated by transferring through solutions of increasing alcohol concentration (30%, 50%, 70%, 90%, and 100%) for 15 min at room temperature followed by acetone for 15 min. Resin (Epon 812 with DDSA and MNA) infiltration was carried out according to the following protocol: Resin-acetone (1:3), resin-acetone (1:1), resin-acetone (3:1) at room temperature for 1 h, and resin alone for 12 h; and resin with a DMP 30 catalysator at 37 °C for 24 h and at 60 °C for 48 h. Hardened blocks were cut on the Leica UC7 (Leica) microtome into 70 nm slices, contrasted by uranyl acetate (30 min) and lead citrate (5 min) and then analyzed using a transmission electron microscope (JEM-1011, Jeol, Akishima, Tokyo, Japan).

### 3.8. Western Blotting

Approximately 50 heads of 3- to 5-day-old adults of each genotype were dissected and homogenized in 8 M urea solution. Equal amounts of samples from the supernatants were preincubated with sample buffer (deionized water, 0.5 M Tris-HCl, glycerol, 10% SDS, 0.5% bromphenol blue, DTT) for 5 min at 95 °C and separated in a 4–12% (*w*/*v*) acrylamide Bis/Tris SDS-PAGE gel using the vertical electrophoretic chamber Mini-Protean Tetra (Bio-Rad). Proteins were transferred from the gel to the PVDF membrane (Immobilon-P Membrane,Millipore, Burlington, MA, USA) using electroblotting (Mini Trans-Blot Modul, Bio-Rad), blocked in BlockPro blocking buffer (Visual Protein Biotechnology Corporation, Taiwan), and incubated with anti–GSK3 beta primary antibodies (1:300; ab18893, Abcam, Cambridge, Great Britain) for one hour. Bound antibodies were detected with goat anti–rabbit secondary antibodies conjugated with alkaline phosphatase (1:20000; A3687, Sigma). Prior to visualization, the membranes were incubated in the alkaline CDP buffer for 5 min and then in the Immun-Star AP- Substrate (Bio-Rad) for 7 min. After scanning, the relative intensity quantification of each band was evaluated with Image Lab software (Bio-Rad). Three independent protein extractions (biological replicates) per genotype were made.

### 3.9. Statistical Analyses

To compare control and mutant genotypes, Student’s *t*-test and the nonparametric, distribution-free Kruskal-Wallis test were used for analyses of *sgg* transcript amounts; locomotion; and numbers of large boutons, satellite boutons, active zones, and mitochondria in NMJs. These two tests gave consistent results, so only the results of the Kruskal-Wallis test are reported here. Standard descriptive statistical analysis of lifespan [56,57] was performed to determine the mean lifespan and its accompanying variances, standard deviations, and standard errors; the median, minimum, and maximum lifespans; and the lifespans of the lower and upper quartiles and the 10 and 90 percentiles (Tables S1–S5). Survival curves were estimated using the Kaplan–Meier procedure. The nonparametric, distribution-free Mann-Whitney test and Kolmogorov-Smirnov test were used to evaluate the statistical significance of the difference between the survival curves.

## 4. Conclusions

Understanding the genetic and molecular basis of pathological changes of the nervous system underlying accelerated aging and shortening of lifespan are important for the development of evidence-based recommendations for life extension. GSK3 is an important enzyme governing numerous regulatory pathways and metabolic processes via interaction with InR/TOR, Wnt, JNK, and other signaling cascades [1,2]. GSK3 is specifically important in the nervous system, where it is involved in the control of synaptic development, structure, and function due to regulation of axonal size, the microtubule cytoskeleton, and neurotransmitter release [7,8,9,10]. Here, we demonstrated GSK involvement in the regulation of mitochondria number, intracellular distribution and structure, and control of synaptic activity. We showed that GSK3 affected the axonal distribution of the microtubule-associated protein, Futsch, thus confirming the role of protein kinases in governing the architecture of the microtubule cytoskeleton. We also demonstrated that GSK3 overexpression caused signs of neurodegeneration in the brains of young individuals. All these findings certified that GSK3 misexpression is crucially important for the structural and functional integrity of the nervous system. The main GSK3 isoform is responsible for the normal operation of neurons.

It is generally clear that the structural and functional integrity of the nervous system, in turn, is important for ensuring a long life. Here, we showed that pathological changes in neurons caused by aberrations in GSK3 expression in the nervous system were paralleled by a rapid decline in survival and shortening lifespan. Our data also demonstrated that GSK3 misexpression in other tissues affected lifespan in a transcript-, stage-, and tissue-specific mode. We proved that some minor *shaggy* transcripts are functional and, moreover, may have the specificity required for a particular function. This result allowed us to lift the veil on the complexity of the gene structure and expression strategy. Most of the *Drosophila* genes have multiple annotated transcripts and proteins (https://flybase.org), and there are not many examples showing whether the alleged complexity of the structural organization of a gene is in line with reality and why such a sophisticated structure is needed. Of particular interest to us was the fact that the embryonic overexpression of one of the minor transcripts was enough to cause an increase in adult lifespan. This fact underscored the role of gene expression early in life for lifespan control and the existence of carry-over mechanisms of an epigenetic or other nature underlying this effect.

Altogether, our results demonstrated the key role of GSK3 in ensuring the link between the pathology of neurons and lifespan. The data presented in this article indicated how disentangling expression strategy and general gene biology might eventually provide a selective approach to the choice of potential drugs and therapies based on an understanding of the molecular mechanisms underlying the development of pathologies shortening lifespan. Obviously, treatment should be aimed at suppressing or enhancing the expression of certain gene transcripts in certain tissues. Our data allowed us to come closer to understanding the characteristics of *shaggy* gene expression, which are important for the search for potential drugs that specifically affect the pathologies of the nervous system and aging caused by the GSK3 malfunction.

## Supplementary Materials

Supplementary Materials can be found at https://www.mdpi.com/1422-0067/20/9/2200/s1.
