# Supplementary material for: Disordered Expression of shaggy, the Drosophila Gene Encoding a Serine-Threonine Protein Kinase GSK3, Affects the Lifespan in a Transcript-, Stage-, and Tissue-Specific Manner
Source: Int J Mol Sci. 2019 May 4;20(9):2200. doi: 10.3390/ijms20092200 (PMC6540023; doi:10.3390/ijms20092200)
Supplement: Supplementary file 1 [file ijms-20-02200-s001.zip › Supplementary Material/Supplementary Figures 1, 2, 3.pdf]

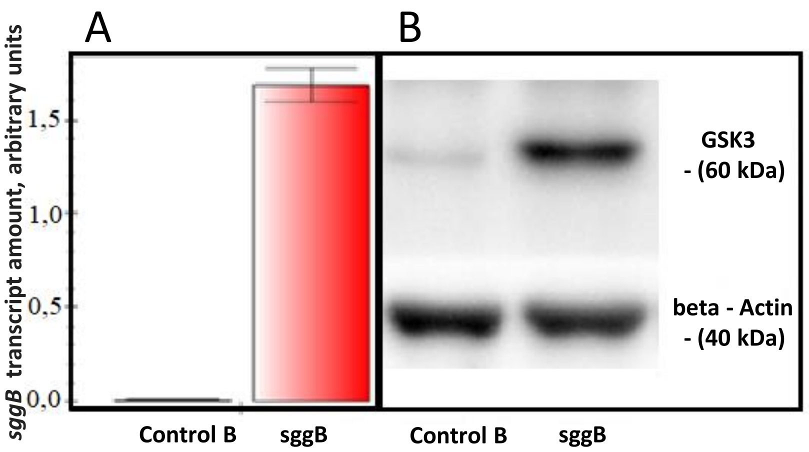

Figure S1. Overexpression of *sgg-RB* (A) and GSK3 (B) in the nervous system measured by real-time RT-qPCR and Western blotting, respectively. Control B, sggB denote hybrid genotypes obtained as a result of crossing the corresponding lines with the driver line D4 inducing the lifelong expression of the transgenic construct in the nervous system. A full description of genotypes is given in the Materials and Methods section.

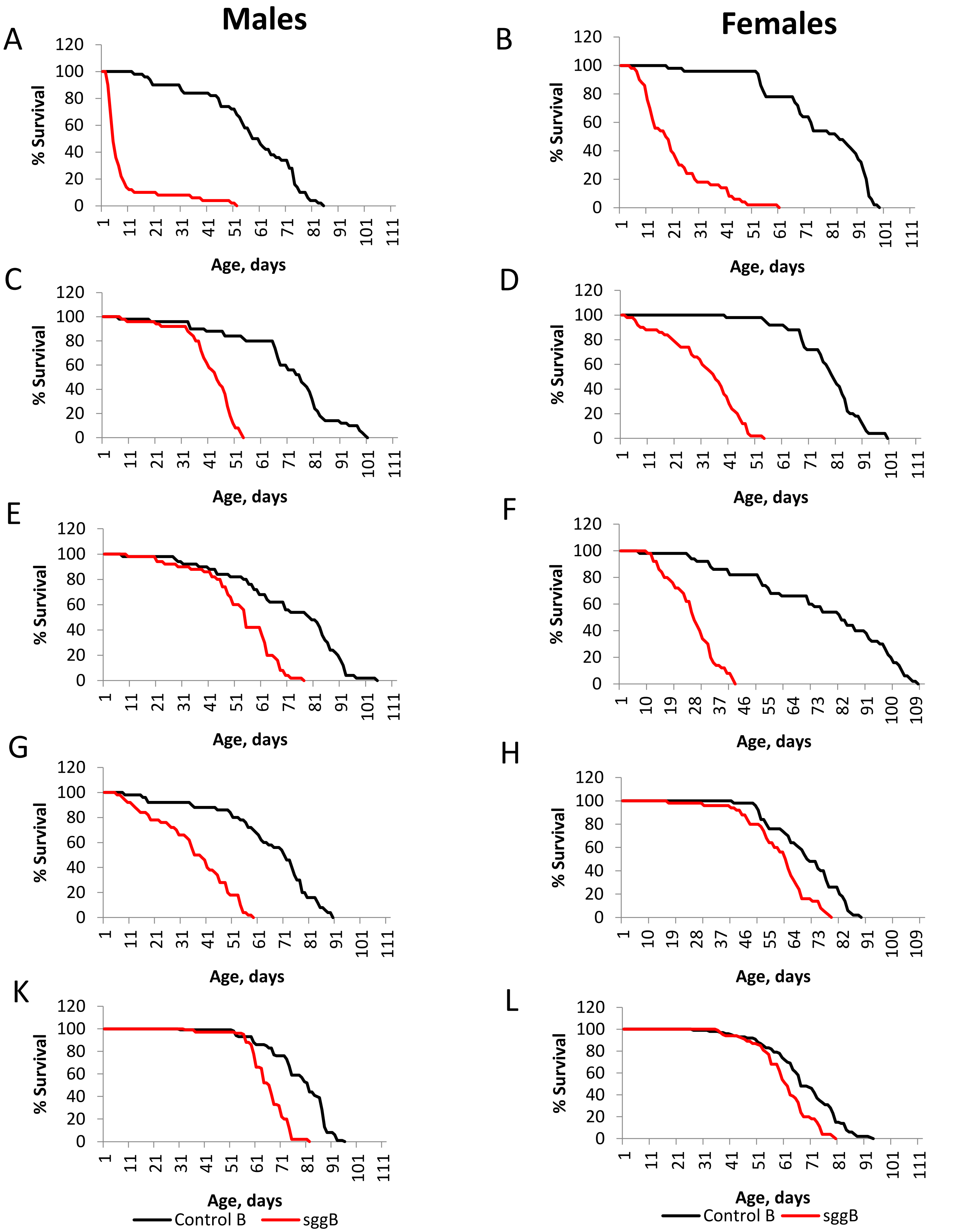

Figure S2. Effects of *sgg-RB* overexpression in peptidergic (A, B), glutamatergic (C, D), cholinergic (E, F), motor (G, H) and GABAergic (I, J) neurons on male (A, C, E, G, I) and female (B, D, F, H, J) lifespans. Control B, sggB denote hybrid genotypes obtained as a result of crossing the corresponding lines with the driver lines D9, D10, D11, D12 or D13. A full description of genotypes is given in the Materials and Methods section.

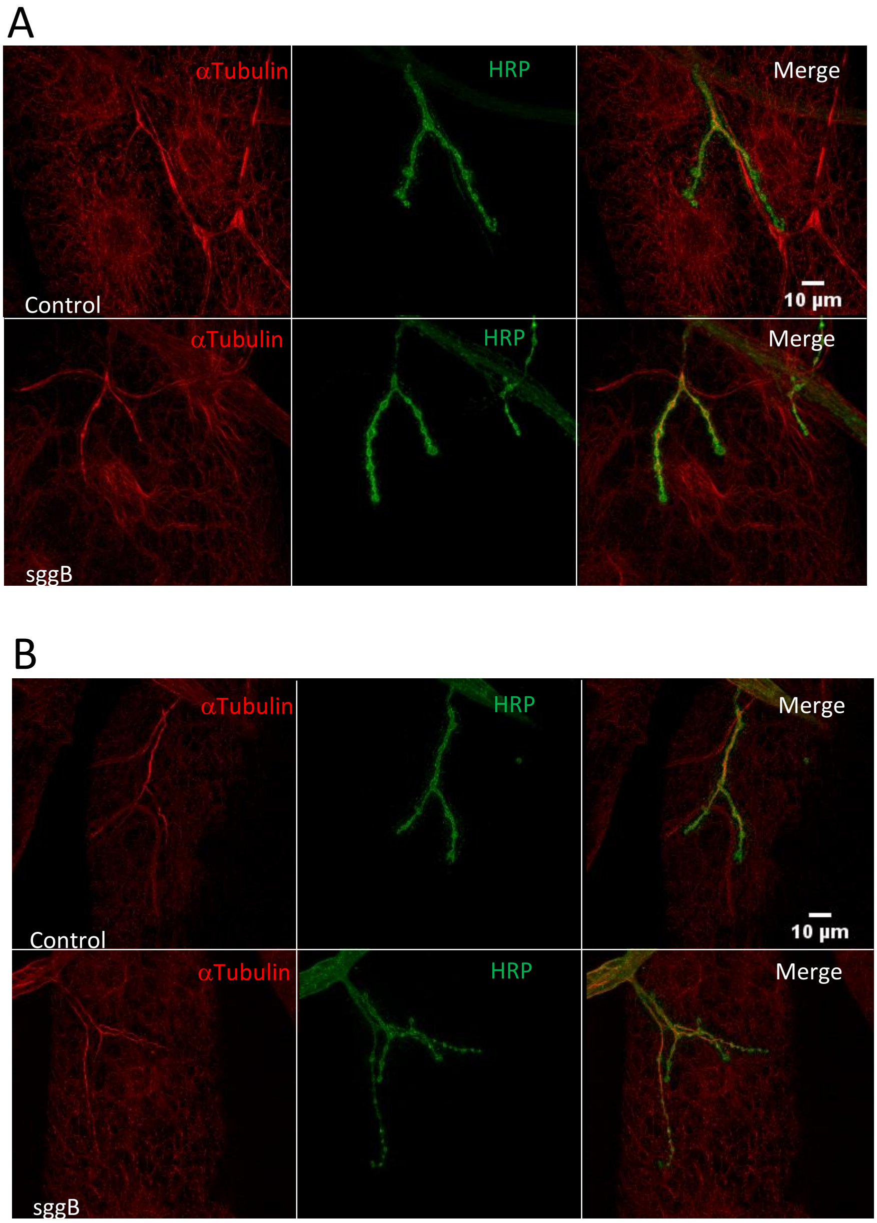

Figure S3. Distribution of tubulin in NMJs of third-instar female larvae with panneuronal *sgg-RB* overexpression (A) and *sgg-RB* overexpression in motor neurons (B). Control B, sggB denote hybrid genotypes obtained as a result of crossing the corresponding lines with the driver line D4 inducing the expression of transgenic constructs in the nervous system or the driver line D12 inducing the expression of transgenic constructs in motor neurons. A full description of genotypes is given in the Materials and Methods section. Representative confocal images of NMJs (muscle 4, hemi-segment 3-4) stained for tubulin (red) and neural membranes (HRP, green). Bar=10 µm.
