# Supplementary material for: Disordered Expression of shaggy, the Drosophila Gene Encoding a Serine-Threonine Protein Kinase GSK3, Affects the Lifespan in a Transcript-, Stage-, and Tissue-Specific Manner
Source: Int J Mol Sci. 2019 May 4;20(9):2200. doi: 10.3390/ijms20092200 (PMC6540023; doi:10.3390/ijms20092200)
Supplement: Supplementary file 1 [file ijms-20-02200-s001.zip › Supplementary Material/Table S1.docx]

Table S1. Embryo. Distributive statistics of the lifespan of transgenic flies with additional copies of *shaggy* and *shaggy* RNAi knockdown.

| Effects | Sex | Genotype | N | Mean | Median | Minimum | Maximum | Lower Quartile | Upper Quartile | Percentile 10 | Percentile 90 | Variance | Standard Deviation | Standard Error | P values for comparisons with control genotype | |
| --- | --- | --- | --- | --- | --- | --- | --- | --- | --- | --- | --- | --- | --- | --- | --- | --- |
|  |  |  |  |  |  |  |  |  |  |  |  |  |  |  | Mann-Whitney Test | Kolmogorov-Smirnov Test |
| *sgg* *RA* overexpression | ♂ | Control | 100 | 64.6 | 65.0 | 23.0 | 101.0 | 58.0 | 71.5 | 47.0 | 82.0 | 229.2 | 15.1 | 1.5 |  |  |
|  |  | Mutant | 100 | 65.0 | 70.0 | 10.0 | 100.0 | 63.0 | 72.0 | 43.5 | 80.5 | 281.8 | 16.8 | 1.7 | p = 0.1671 | p > 0.10 |
|  | ♀ | Control | 100 | 65.3 | 68.0 | 27.0 | 89.0 | 58.5 | 73.5 | 49.5 | 79.0 | 148.6 | 12.2 | 1.2 |  |  |
|  |  | Mutant | 100 | 62.8 | 63.0 | 4.0 | 97.0 | 55.5 | 72.0 | 45.0 | 81.5 | 252.8 | 15.9 | 1.6 | p = 0.1846 | p < 0.10 |
| *sgg RB* overexpression | ♂ | Control | 100 | Not analyzed | | | | | | | | | | | | |
|  |  | Mutant | 100 | Lethal | | | | | | | | | | | | |
|  | ♀ | Control | 100 | Not analyzed | | | | | | | | | | | | |
|  |  | Mutant | 100 | Lethal | | | | | | | | | | | | |
| *sgg RG* overexpression | ♂ | Control | 100 | 64.6 | 65.0 | 23.0 | 101.0 | 58.0 | 71.5 | 47.0 | 82.0 | 229.2 | 15.1 | 1.5 |  |  |
|  |  | Mutant | 100 | 61.1 | 65.0 | 7.0 | 98.0 | 57.0 | 77.0 | 23.0 | 84.0 | 482.4 | 22.0 | 2.2 | p = 0.7378 | p > 0.10 |
|  | ♀ | Control | 100 | 65.3 | 68.0 | 27.0 | 89.0 | 58.5 | 73.5 | 49.5 | 79.0 | 148.6 | 12.2 | 1.2 |  |  |
|  |  | Mutant | 100 | 64.9 | 73.0 | 4.0 | 100.0 | 62.0 | 79.5 | 19.5 | 87.0 | 549.8 | 23.4 | 2.3 | p = 0.0582 | **p < 0.01** |
| *sgg RO* overexpression | ♂ | Control | 100 | 64.6 | 65.0 | 23.0 | 101.0 | 58.0 | 71.5 | 47.0 | 82.0 | 229.2 | 15.1 | 1.5 |  |  |
|  |  | Mutant | 100 | 72.0 | 78.0 | 6.0 | 101.0 | 61.5 | 83.5 | 53.0 | 88.5 | 301.0 | 17.4 | 1.7 | **P < 0.0001** | **P < 0.001** |
|  |  | Control | 100 | 68.5 | 72.0 | 4.0 | 90.0 | 61.5 | 79.0 | 53.5 | 82.0 | 233.4 | 15.3 | 1.5 |  |  |
|  |  | Mutant | 100 | 72.5 | 75.0 | 21.0 | 93.0 | 64.5 | 82.0 | 57.5 | 86.0 | 153.8 | 12.4 | 1.2 | **p = 0.0476** | p > 0.10 |
|  | ♀ | Control | 100 | 65.3 | 68.0 | 27.0 | 89.0 | 58.5 | 73.5 | 49.5 | 79.0 | 148.6 | 12.2 | 1.2 |  |  |
|  |  | Mutant | 100 | 60.1 | 64.0 | 6.0 | 84.0 | 52.0 | 70.0 | 40.5 | 77.0 | 212.8 | 14.6 | 1.5 | **p = 0.0099** | p < 0.05 |
|  |  | Control | 100 | 65.3 | 64.0 | 28.0 | 96.0 | 57.5 | 73.0 | 49.5 | 82.5 | 175.2 | 13.2 | 1.3 |  |  |
|  |  | Mutant | 100 | 61.1 | 61.0 | 42.0 | 87.0 | 53.5 | 69.0 | 46.0 | 76.0 | 122.8 | 11.1 | 1.1 | **p = 0.0136** | p < 0.10 |
| Strong *sgg* knockdown | ♂ | Control | 100 | Not analyzed | | | | | | | | | | | | |
|  |  | Mutant | 100 | Lethal | | | | | | | | | | | | |
|  | ♀ | Control | 100 | Not analyzed | | | | | | | | | | | | |
|  |  | Mutant | 100 | Lethal | | | | | | | | | | | | |
| Weak *sgg* knockdown | ♂ | Control | 100 | 84.0 | 91.0 | 3.0 | 115.0 | 76.5 | 95.0 | 64.0 | 102.0 | 395.8 | 19.9 | 2.0 |  |  |
|  |  | Mutant | 100 | 64.3 | 71.0 | 20.0 | 90.0 | 51.5 | 75.0 | 44.5 | 82.0 | 256.2 | 16.0 | 1.6 | **P < 0.0001** | **P < 0.001** |
|  | ♀ | Control | 100 | 80.3 | 83.5 | 43.0 | 99.0 | 71.0 | 90.0 | 62.0 | 94.0 | 160.1 | 12.7 | 1.3 |  |  |
|  |  | Mutant | 100 | 36.8 | 40.5 | 3.0 | 59.0 | 30.0 | 45.5 | 18.5 | 49.0 | 150.0 | 12.2 | 1.2 | **P < 0.0001** | **P < 0.001** |

Different pairs Control-Mutant of the same genotype and sex represent the results of independent experiments. Full description of genotypes is given in the Materials and Methods section. Significant (after Bonferroni corrections when appropriate) P-values are in bold case.
