# Supplementary material for: Disordered Expression of shaggy, the Drosophila Gene Encoding a Serine-Threonine Protein Kinase GSK3, Affects the Lifespan in a Transcript-, Stage-, and Tissue-Specific Manner
Source: Int J Mol Sci. 2019 May 4;20(9):2200. doi: 10.3390/ijms20092200 (PMC6540023; doi:10.3390/ijms20092200)
Supplement: Supplementary file 1 [file ijms-20-02200-s001.zip › Supplementary Material/Table S2.docx]

| Effects | Sex | Genotype | N | Mean | Median | Minimum | | Maximum | | Lower Quartile | Upper Quartile | | Percentile 10 | | Percentile 90 | Variance | | Standard Deviation | | Standard Error | | P values for comparisons with control genotype | | |
| --- | --- | --- | --- | --- | --- | --- | --- | --- | --- | --- | --- | --- | --- | --- | --- | --- | --- | --- | --- | --- | --- | --- | --- | --- |
|  |  |  |  |  |  |  |  |  |  |  |  |  |  |  |  |  |  |  |  |  |  | Mann-Whitney Test | Kolmogorov-Smirnov Test | |
| *sgg RA* overexpression | ♂ | Control | 100 | 79.1 | 78.5 | 42.0 | | 107.0 | | 71.5 | 87.0 | | 61.0 | | 97.0 | 177.1 | | 13.3 | | 1.3 | |  |  | |
|  |  | Mutant | 100 | 75.1 | 75.0 | 7.0 | | 102.0 | | 67.5 | 88.5 | | 52.5 | | 96.0 | 372.0 | | 19.3 | | 1.9 | | P = 0.2802 | P > 0.10 | |
|  | ♀ | Control | 100 | 84.2 | 84.0 | 44.0 | | 113.0 | | 76.0 | 94.0 | | 68.5 | | 101.0 | 178.1 | | 13.3 | | 1.3 | |  |  | |
|  |  | Mutant | 100 | 79.4 | 82.5 | 3.0 | | 118.0 | | 74.0 | 93.0 | | 55.0 | | 99.5 | 406.5 | | 20.2 | | 2.0 | | P = 0.2834 | P > 0.10 | |
| *sgg* *RB* overexpression | ♂ | Control | 100 | Not analyzed | | | | | | | | | | | | | | | | | | | | |
|  |  | Mutant | 100 | Lethal | | | | | | | | | | | | | | | | | | | | |
|  | ♀ | Control | 100 | Not analyzed | | | | | | | | | | | | | | | | | | | | |
|  |  | Mutant | 100 | Lethal | | | | | | | | | | | | | | | | | | | | |
| *sgg RG* overexpression | ♂ | Control | 100 | 79.1 | 78.5 | 42.0 | | 107.0 | | 71.5 | 87.0 | | 61.0 | | 97.0 | 177.1 | | 13.3 | | 1.3 | |  |  | |
|  |  | Mutant | 100 | 78.5 | 82.0 | 12.0 | | 113.0 | | 71.5 | 93.5 | | 48.0 | | 104.0 | 482.7 | | 22.0 | | 2.2 | | P = 0.2538 | P > 0.10 | |
|  | ♀ | Control | 100 | 84.2 | 84.0 | 44.0 | | 113.0 | | 76.0 | 94.0 | | 68.5 | | 101.0 | 178.1 | | 13.3 | | 1.3 | |  |  | |
|  |  | Mutant | 100 | 84.0 | 87.0 | 31.0 | | 108.0 | | 74.0 | 94.0 | | 68.0 | | 99.0 | 209.6 | | 14.5 | | 1.4 | | P = 0.8632 | P > 0.10 | |
| *sgg RO* overexpression | ♂ | Control | 100 | 79.1 | 78.5 | 42.0 | | 107.0 | | 71.5 | 87.0 | | 61.0 | | 97.0 | 177.1 | | 13.3 | | 1.3 | |  |  | |
|  |  | Mutant | 100 | 77.5 | 82.0 | 9.0 | | 99.0 | | 75.0 | 88.0 | | 59.0 | | 92.0 | 273.2 | | 16.5 | | 1.7 | | P = 0.6389 | P > 0.10 | |
|  | ♀ | Control | 100 | 84.2 | 84.0 | 44.0 | | 113.0 | | 76.0 | 94.0 | | 68.5 | | 101.0 | 178.1 | | 13.3 | | 1.3 | |  |  | |
|  |  | Mutant | 100 | 82.0 | 84.0 | 19.0 | | 109.0 | | 77.0 | 94.0 | | 63.5 | | 101.0 | 276.3 | | 16.6 | | 1.7 | | P = 0.7314 | P > 0.10 | |
| Strong *sgg* knockdown | ♂ | Control | 100 | Not analyzed | | | | | | | | | | | | | | | | | | | | |
|  |  | Mutant | 100 | Lethal | | | | | | | | | | | | | | | | | | | | |
|  | ♀ | Control | 100 | Not analyzed | | | | | | | | | | | | | | | | | | | | |
|  |  | Mutant | 100 | Lethal | | | | | | | | | | | | | | | | | | | | |
| Weak *sgg* knockdown | ♂ | Control | 100 | 90.5 | 94.0 | | 29.0 | | 116.0 | 82.0 | | 102.5 | | 69.0 | 108.0 | | 279.2 | | 16.7 | | 1.7 |  | |  |
|  |  | Mutant | 100 | 82.2 | 84.5 | | 35.0 | | 106.0 | 76.0 | | 92.0 | | 66.0 | 97.5 | | 156.8 | | 12.5 | | 1.3 | **P < 0.0001** | | **P < 0.001** |
|  | ♀ | Control | 100 | 77.6 | 82.5 | | 22.0 | | 105.0 | 69.0 | | 91.0 | | 49.0 | 97.0 | | 341.5 | | 18.5 | | 1.8 |  | |  |
|  |  | Mutant | 100 | 80.5 | 81.0 | | 9.0 | | 100.0 | 74.0 | | 89.5 | | 65.5 | 94.5 | | 180.3 | | 13.4 | | 1.3 | P = 0.5917 | | P > 0.10 |

Table S2. The fat body. Distributive statistics of the lifespan of transgenic flies with additional copies of *shaggy* and *shaggy* RNAi knockdown.

Different pairs Control-Mutant of the same genotype and sex represent the results of independent experiments. Full description of genotypes is given in the Materials and Methods section. Significant P-values are in bold case.
