# Supplementary material for: Disordered Expression of shaggy, the Drosophila Gene Encoding a Serine-Threonine Protein Kinase GSK3, Affects the Lifespan in a Transcript-, Stage-, and Tissue-Specific Manner
Source: Int J Mol Sci. 2019 May 4;20(9):2200. doi: 10.3390/ijms20092200 (PMC6540023; doi:10.3390/ijms20092200)
Supplement: Supplementary file 1 [file ijms-20-02200-s001.zip › Supplementary Material/Table S3.docx]

Table S3. Muscles. Distributive statistics of the lifespan of transgenic flies with additional copies of *shaggy* and *shaggy* RNAi knockdown.

| Effects | Sex | Genotype | N | Mean | Median | Minimum | Maximum | Lower Quartile | Upper Quartile | Percentile 10 | Percentile 90 | Variance | Standard Deviation | Standard Error | P values for comparisons with control genotype | |
| --- | --- | --- | --- | --- | --- | --- | --- | --- | --- | --- | --- | --- | --- | --- | --- | --- |
|  |  |  |  |  |  |  |  |  |  |  |  |  |  |  | Mann-Whitney Test | Kolmogorov-Smirnov Test |
| *sgg* *RA* overexpression | ♂ | Control | 100 | 64.1 | 66.0 | 18.0 | 92.0 | 54.0 | 79.0 | 41.0 | 82.0 | 259.7 | 16.1 | 1.6 |  |  |
|  |  | Mutant | 100 | 52.4 | 55.5 | 12.0 | 83.0 | 40.5 | 64.0 | 25.5 | 77.0 | 295.5 | 17.2 | 1.7 | **P < 0.0001** | **P < 0.001** |
|  |  | Control | 100 | 61.7 | 68.0 | 19.0 | 89.0 | 53.0 | 74.0 | 22.0 | 74.0 | 310.9 | 17.6 | 1.8 |  |  |
|  |  | Mutant | 100 | 55.0 | 67.0 | 12.0 | 97.0 | 41.5 | 68.0 | 26.5 | 74.0 | 377.9 | 19.4 | 1.9 | **P = 0.0058** | **P < 0.005** |
|  | ♀ | Control | 100 | 69.9 | 74.0 | 17.0 | 93.0 | 66.5 | 80.0 | 42.5 | 86.0 | 281.5 | 16.8 | 1.7 |  |  |
|  |  | Mutant | 100 | 60.0 | 62.5 | 13.0 | 93.0 | 45.5 | 76.0 | 31.0 | 82.5 | 372.9 | 19.3 | 1.9 | **P = 0.0002** | **P < 0.001** |
|  |  | Control | 100 | 69.3 | 73.0 | 19.0 | 96.0 | 66.0 | 79.5 | 46.5 | 86.5 | 293.2 | 17.1 | 1.7 |  |  |
|  |  | Mutant | 100 | 64.3 | 70.0 | 13.0 | 97.0 | 55.5 | 80.0 | 19.0 | 86.5 | 470.8 | 21.7 | 2.2 | P = 0.1384 | P > 0.10 |
| *sgg RB* overexpression | ♂ | Control | 100 | 62.9 | 64.0 | 11.0 | 91.0 | 54.0 | 73.0 | 47.0 | 80.5 | 213.3 | 14.6 | 1.5 |  |  |
|  |  | Mutant | 100 | 57.1 | 57.0 | 4.0 | 94.0 | 46.5 | 65.0 | 40.5 | 74.0 | 223.6 | 15.0 | 1.5 | **P = 0.0012** | **P < 0.025** |
|  | ♀ | Control | 100 | 68.8 | 71.0 | 18.0 | 86.0 | 67.0 | 73.5 | 56.0 | 75.0 | 97.1 | 9.9 | 1.0 |  |  |
|  |  | Mutant | 100 | 65.8 | 68.0 | 24.0 | 88.0 | 61.0 | 74.0 | 45.5 | 82.0 | 171.6 | 13.1 | 1.3 | **P = 0.0325** | **P < 0.005** |
| *sgg RG* overexpression | ♂ | Control | 100 | 64.1 | 66.0 | 18.0 | 92.0 | 54.0 | 79.0 | 41.0 | 82.0 | 259.7 | 16.1 | 1.6 |  |  |
|  |  | Mutant | 100 | 62.0 | 68.0 | 13.0 | 84.0 | 50.5 | 73.0 | 43.5 | 79.0 | 247.8 | 15.7 | 1.6 | P = 0.3147 | P > 0.10 |
|  | ♀ | Control | 100 | 69.9 | 74.0 | 17.0 | 93.0 | 66.5 | 80.0 | 42.5 | 86.0 | 281.5 | 16.8 | 1.7 |  |  |
|  |  | Mutant | 100 | 68.8 | 74.0 | 6.0 | 98.0 | 61.0 | 79.5 | 39.5 | 85.0 | 305.8 | 17.5 | 1.7 | P = 0.5926 | P > 0.10 |
| *sgg RO* overexpression | ♂ | Control | 100 | 64.1 | 66.0 | 18.0 | 92.0 | 54.0 | 79.0 | 41.0 | 82.0 | 259.7 | 16.1 | 1.6 |  |  |
|  |  | Mutant | 100 | 61.4 | 64.0 | 28.0 | 87.0 | 51.0 | 75.0 | 34.5 | 81.0 | 271.9 | 16.5 | 1.6 | P = 0.2191 | P > 0.10 |
|  | ♀ | Control | 100 | 69.9 | 74.0 | 17.0 | 93.0 | 66.5 | 80.0 | 42.5 | 86.0 | 281.5 | 16.8 | 1.7 |  |  |
|  |  | Mutant | 100 | 72.2 | 74.0 | 11.0 | 93.0 | 69.0 | 81.5 | 51.5 | 88.0 | 217.8 | 14.8 | 1.5 | P = 0.5212 | P > 0.10 |
| Strong *sgg* knockdown | ♂ | Control | 100 | 72.9 | 73.0 | 33.0 | 96.0 | 65.0 | 81.0 | 56.5 | 91.0 | 151.3 | 12.3 | 1.2 |  |  |
|  |  | Mutant | 100 | 45.9 | 45.0 | 17.0 | 76.0 | 36.0 | 55.0 | 31.0 | 62.0 | 150.1 | 12.3 | 1.2 | **P < 0.0001** | **P < 0.001** |
|  | ♀ | Control | 100 | 78.5 | 83.0 | 16.0 | 99.0 | 73.0 | 89.0 | 60.5 | 93.5 | 301.0 | 17.3 | 1.7 |  |  |
|  |  | Mutant | 100 | 62.2 | 65.0 | 9.0 | 77.0 | 58.0 | 71.0 | 48.5 | 72.0 | 141.6 | 11.9 | 1.2 | **P < 0.0001** | **P < 0.001** |
| Weak *sgg* knockdown | ♂ | Control | 100 | 66.5 | 66.5 | 5.0 | 99.0 | 60.0 | 78.0 | 52.0 | 84.0 | 260.7 | 16.1 | 1.6 |  |  |
|  |  | Mutant | 100 | 55.0 | 56.0 | 16.0 | 75.0 | 49.0 | 63.0 | 40.0 | 70.0 | 149.6 | 12.2 | 1.2 | **P < 0.0011** | **P < 0.001** |
|  | ♀ | Control | 100 | 81.3 | 85.0 | 12.0 | 100.0 | 76.5 | 90.0 | 61.5 | 94.0 | 191.5 | 13.8 | 1.4 |  |  |
|  |  | Mutant | 100 | 72.7 | 74.0 | 41.0 | 93.0 | 65.0 | 79.0 | 59.5 | 86.5 | 110.2 | 10.5 | 1.0 | **P < 0.0001** | **P < 0.001** |

Different pairs Control-Mutant of the same genotype and sex represent the results of independent experiments. Full description of genotypes is given in the Materials and Methods section. Significant P-values are in bold case.
