# Supplementary material for: Disordered Expression of shaggy, the Drosophila Gene Encoding a Serine-Threonine Protein Kinase GSK3, Affects the Lifespan in a Transcript-, Stage-, and Tissue-Specific Manner
Source: Int J Mol Sci. 2019 May 4;20(9):2200. doi: 10.3390/ijms20092200 (PMC6540023; doi:10.3390/ijms20092200)
Supplement: Supplementary file 1 [file ijms-20-02200-s001.zip › Supplementary Material/Table S4.docx]

Table S4. The nervous system. Distributive statistics of the lifespan of transgenic flies with additional copies of *shaggy* and *shaggy* RNAi knockdown.

| Effects | Sex | Genotype | N | Mean | Median | Minimum | Maximum | Lower Quartile | Upper Quartile | Percentile 10 | Percentile 90 | Variance | Standard Deviation | Standard Error | P values for comparisons with control genotype | |
| --- | --- | --- | --- | --- | --- | --- | --- | --- | --- | --- | --- | --- | --- | --- | --- | --- |
|  |  |  |  |  |  |  |  |  |  |  |  |  |  |  | Mann-Whitney Test | Kolmogorov-Smirnov Test |
| *sgg* *RA* panneuronal overexpression | ♂ | Control | 100 | 61.1 | 63.0 | 10.0 | 91.0 | 55.0 | 69.0 | 44.5 | 78.0 | 241.5 | 15.5 | 1.6 |  |  |
|  |  | Mutant | 100 | 63.5 | 66.0 | 10.0 | 94.0 | 59.0 | 72.0 | 49.0 | 77.0 | 211.5 | 14.5 | 1.5 | P = 0.2017 | P > 0.10 |
|  | ♀ | Control | 100 | 73.0 | 75.0 | 25.0 | 86.0 | 70.0 | 79.0 | 61.0 | 82.0 | 102.9 | 10.1 | 1.0 |  |  |
|  |  | Mutant | 100 | 70.7 | 77.0 | 4.0 | 90.0 | 69.0 | 79.5 | 49.0 | 84.0 | 333.7 | 18.3 | 1.8 | P = 0.5134 | P > 0.10 |
| *sgg RB* panneuronal overexpression | ♂ | Control | 100 | 69.1 | 75.0 | 8.0 | 92.0 | 58.5 | 81.5 | 44.5 | 90.0 | 325.5 | 18.0 | 1.8 |  |  |
|  |  | Mutant | 100 | 2.3 | 2.0 | 1.0 | 5.0 | 1.0 | 3.0 | 1.0 | 4.0 | 1.3 | 1.1 | 0.1 | **P < 0.0001** | **P < 0.001** |
|  |  | Control | 100 | 80.9 | 83.0 | 4.0 | 108.0 | 76.0 | 94.0 | 59.0 | 101.0 | 374.1 | 19.3 | 1.9 |  |  |
|  |  | Mutant | 100 | 1.4 | 1.0 | 1.0 | 4.0 | 1.0 | 2.0 | 1.0 | 2.0 | 0.4 | 0.6 | 0.1 | **P < 0.0001** | **P < 0.001** |
|  | ♀ | Control | 100 | 73.1 | 77.0 | 7.0 | 96.0 | 70.0 | 82.0 | 58.5 | 86.0 | 260.4 | 16.1 | 1.6 |  |  |
|  |  | Mutant | 100 | 6.5 | 5.0 | 1.0 | 23.0 | 3.5 | 9.0 | 2.0 | 11.0 | 17.0 | 4.1 | 0.4 | **P < 0.0001** | **P < 0.001** |
|  |  | Control | 100 | 88.8 | 94.0 | 8.0 | 111.0 | 81.0 | 100.0 | 67.5 | 101.0 | 244.9 | 15.6 | 1.6 |  |  |
|  |  | Mutant | 100 | 3.7 | 3.0 | 1.0 | 22.0 | 2.0 | 4.0 | 1.0 | 6.0 | 7.8 | 2.8 | 0.3 | **P < 0.0001** | **P < 0.001** |
| *sgg RB* overexpression in the embryonic CNS | ♂ | Control | 100 | 70.6 | 79.0 | 6.0 | 99.0 | 56.0 | 87.0 | 37.0 | 90.0 | 446.2 | 21.1 | 3.0 |  |  |
|  |  | Mutant | 100 | 42.6 | 47.5 | 1.0 | 70.0 | 27.0 | 56.0 | 10.0 | 62.0 | 402.0 | 20.1 | 2.8 | **P < 0.0001** | **P < 0.001** |
|  | ♀ | Control | 100 | 79.8 | 81.5 | 41.0 | 102.0 | 77.0 | 88.0 | 64.5 | 92.0 | 142.2 | 11.9 | 1.7 |  |  |
|  |  | Mutant | 100 | 63.7 | 69.5 | 3.0 | 102.0 | 54.0 | 83.0 | 28.0 | 89.0 | 658.6 | 25.7 | 3.6 | **P = 0.0008** | **P < 0.001** |
| *sgg RB* overexpression in the adult nervous system, increasing with age | ♂ | Control | 100 | 81.2 | 86.0 | 6.0 | 104.0 | 78.0 | 94.0 | 53.5 | 99.0 | 430.4 | 20.7 | 2.1 |  |  |
|  |  | Mutant | 100 | 35.7 | 33.0 | 9.0 | 63.0 | 32.0 | 38.0 | 27.0 | 49.0 | 85.2 | 9.2 | 0.9 | **P < 0.0001** | **P < 0.001** |
|  | ♀ | Control | 100 | 65.4 | 67.0 | 8.0 | 93.0 | 59.0 | 75.5 | 48.0 | 81.0 | 214.4 | 14.6 | 1.5 |  |  |
|  |  | Mutant | 100 | 47.8 | 42.0 | 21.0 | 97.0 | 35.5 | 55.5 | 30.5 | 76.5 | 289.6 | 17.0 | 1.7 | **P < 0.0001** | **P < 0.001** |
| *sgg RG* panneuronal overexpression | ♂ | Control | 100 | 61.1 | 63.0 | 10.0 | 91.0 | 55.0 | 69.0 | 44.5 | 78.0 | 241.5 | 15.5 | 1.6 |  |  |
|  |  | Mutant | 100 | 56.6 | 58.5 | 4.0 | 93.0 | 50.0 | 68.0 | 30.0 | 82.0 | 384.1 | 19.6 | 2.0 | P = 0.0559 | P < 0.05 |
|  | ♀ | Control | 100 | 73.0 | 75.0 | 25.0 | 86.0 | 70.0 | 79.0 | 61.0 | 82.0 | 102.9 | 10.1 | 1.0 |  |  |
|  |  | Mutant | 100 | 70.9 | 77.0 | 5.0 | 91.0 | 67.0 | 81.0 | 42.5 | 87.0 | 319.8 | 17.9 | 1.8 | P = 0.3653 | P > 0.10 |
| *sgg RO* panneuronal overexpression | ♂ | Control | 100 | 61.1 | 63.0 | 10.0 | 91.0 | 55.0 | 69.0 | 44.5 | 78.0 | 241.5 | 15.5 | 1.6 |  |  |
|  |  | Mutant | 100 | 57.7 | 62.0 | 11.0 | 84.0 | 52.0 | 68.5 | 33.5 | 75.0 | 276.0 | 16.6 | 1.7 | P = 0.1557 | P > 0.10 |
|  | ♀ | Control | 100 | 73.0 | 75.0 | 25.0 | 86.0 | 70.0 | 79.0 | 61.0 | 82.0 | 102.9 | 10.1 | 1.0 |  |  |
|  |  | Mutant | 100 | \| 74.9 \| 78.0 \| 17.0 \| 92.0 \| 71.0 \| 82.0 \| 63.0 \| 84.5 \| 158.4 \| 12.6 \| 1.3 \| \| --- \| --- \| --- \| --- \| --- \| --- \| --- \| --- \| --- \| --- \| --- \| | 78.0 | 17.0 | 92.0 | 71.0 | 82.0 | 63.0 | 84.5 | 158.4 | 12.6 | 1.3 | P = 0.2141 | P < 0.05 |
| Strong panneuronal *sgg* knockdown | ♂ | Control | Not analyzed | | | | | | | | | | | | | |
|  |  | Mutant | Lethal | | | | | | | | | | | | | |
|  | ♀ | Control | Not analyzed | | | | | | | | | | | | | |
|  |  | Mutant | Lethal | | | | | | | | | | | | | |
| Weak panneuronal *sgg* knockdown | ♂ | Control | 100 | 62.1 | 67.0 | 7.0 | 85.0 | 57.5 | 71.0 | 43.0 | 77.5 | 252.8 | 15.9 | 1.6 |  |  |
|  |  | Mutant | 100 | 58.8 | 61.0 | 17.0 | 94.0 | 49.0 | 68.0 | 40.0 | 75.0 | 210.7 | 14.5 | 1.5 | **P = 0.0095** | **P < 0.025** |
|  | ♀ | Control | 100 | 84.4 | 85.0 | 18.0 | 104.0 | 80.5 | 91.0 | 71.5 | 95.5 | 134.5 | 11.6 | 1.2 |  |  |
|  |  | Mutant | 100 | 77.0 | 80.0 | 14.0 | 97.0 | 70.0 | 86.0 | 66.0 | 93.0 | 219.1 | 14.8 | 1.5 | **P < 0.0001** | **P < 0.001** |

Different pairs Control-Mutant of the same genotype and sex represent the results of independent experiments. Full description of genotypes is given in the Materials and Methods section. Significant (after Bonferroni corrections when appropriate) P-values are in bold case.

CNS: Central Nervous System.
