# Supplementary material for: Disordered Expression of shaggy, the Drosophila Gene Encoding a Serine-Threonine Protein Kinase GSK3, Affects the Lifespan in a Transcript-, Stage-, and Tissue-Specific Manner
Source: Int J Mol Sci. 2019 May 4;20(9):2200. doi: 10.3390/ijms20092200 (PMC6540023; doi:10.3390/ijms20092200)
Supplement: Supplementary file 1 [file ijms-20-02200-s001.zip › Supplementary Material/Table S5.docx]

Table S5. Distributive statistics of the lifespan of transgenic flies with *shaggy* *RB* overexpression in different neurons.

| Neurons | Sex | Genotype | N | Mean | Median | Minimum | Maximum | Lower Quartile | Upper Quartile | Percentile 10 | Percentile 90 | Variance | Standard Deviation | Standard Error | P values for comparisons with control genotype | |
| --- | --- | --- | --- | --- | --- | --- | --- | --- | --- | --- | --- | --- | --- | --- | --- | --- |
|  |  |  |  |  |  |  |  |  |  |  |  |  |  |  | Mann-Whitney Test | Kolmogorov-Smirnov Test |
| Dophaminergic (D7 driver) | ♂ | Control | 50 | Not analyzed | | | | | | | | | | | | |
|  |  | Mutant | 50 | Lethal | | | | | | | | | | | | |
|  | ♀ | Control | 50 | Not analyzed | | | | | | | | | | | | |
|  |  | Mutant | 50 | Lethal | | | | | | | | | | | | |
| Dophaminergic (D8 driver) | ♂ | Control | 50 | Not analyzed | | | | | | | | | | | | |
|  |  | Mutant | 50 | Lethal | | | | | | | | | | | | |
|  | ♀ | Control | 50 | Not analyzed | | | | | | | | | | | | |
|  |  | Mutant | 50 | Lethal | | | | | | | | | | | | |
| Peptidergic | ♂ | Control | 50 | 56.4 | 58.5 | 12.0 | 84.0 | 45.0 | 73.0 | 24.5 | 76.5 | 355.9 | 18.9 | 2.7 |  |  |
|  |  | Mutant | 50 | 8.2 | 4.0 | 2.0 | 51.0 | 3.0 | 7.0 | 2.5 | 16.5 | 122.1 | 11.0 | 1.6 | **P < 0.0001** | **P < 0.001** |
|  | ♀ | Control | 50 | 76.3 | 82.5 | 18.0 | 98.0 | 66.0 | 93.0 | 53.0 | 94.0 | 362.0 | 19.0 | 2.7 |  |  |
|  |  | Mutant | 50 | 20.4 | 17.5 | 4.0 | 60.0 | 11.0 | 25.0 | 7.5 | 41.0 | 165.9 | 12.9 | 1.8 | **P < 0.0001** | **P < 0.001** |
| Glatamatergic | ♂ | Control | 50 | 69.7 | 75.0 | 6.0 | 100.0 | 65.0 | 80.0 | 36.0 | 95.0 | 415.5 | 20.4 | 2.9 |  |  |
|  |  | Mutant | 50 | 40.9 | 43.0 | 7.0 | 53.0 | 37.0 | 48.0 | 32.0 | 50.0 | 98.2 | 9.9 | 1.4 | **P < 0.0001** | **P < 0.001** |
|  | ♀ | Control | 50 | 77.1 | 79.0 | 39.0 | 99.0 | 68.0 | 84.0 | 61.5 | 90.5 | 144.1 | 12.0 | 1.7 |  |  |
|  |  | Mutant | 50 | 31.5 | 35.0 | 2.0 | 53.0 | 22.0 | 41.0 | 8.0 | 47.0 | 176.1 | 13.3 | 1.9 | **P < 0.0001** | **P < 0.001** |
| Cholinergic | ♂ | Control | 50 | 70.2 | 78.5 | 7.0 | 104.0 | 57.0 | 86.0 | 38.0 | 92.0 | 475.7 | 21.8 | 3.1 |  |  |
|  |  | Mutant | 50 | 52.1 | 54.0 | 9.0 | 76.0 | 45.0 | 62.0 | 30.5 | 67.0 | 208.2 | 14.4 | 2.0 | **P < 0.0001** | **P < 0.001** |
|  | ♀ | Control | 50 | 73.7 | 81.0 | 7.0 | 109.0 | 52.0 | 98.0 | 33.0 | 103.5 | 734.9 | 27.1 | 3.8 |  |  |
|  |  | Mutant | 50 | 26.6 | 27.0 | 10.0 | 42.0 | 20.0 | 33.0 | 14.0 | 39.0 | 77.8 | 8.8 | 1.2 | **P < 0.0001** | **P < 0.001** |
| Motor | ♂ | Control | 50 | 64.0 | 70.5 | 8.0 | 89.0 | 56.0 | 77.0 | 34.5 | 84.0 | 392.0 | 19.8 | 2.8 |  |  |
|  |  | Mutant | 50 | 35.0 | 36.5 | 5.0 | 58.0 | 25.0 | 48.0 | 11.5 | 53.5 | 230.8 | 15.2 | 2.1 | **P < 0.0001** | **P < 0.001** |
|  | ♀ | Control | 50 | 68.0 | 68.5 | 41.0 | 88.0 | 59.0 | 80.0 | 51.0 | 83.0 | 150.4 | 12.3 | 1.7 |  |  |
|  |  | Mutant | 50 | 58.0 | 60.5 | 17.0 | 77.0 | 52.0 | 65.0 | 44.0 | 73.0 | 139.3 | 11.8 | 1.7 | **P = 0.0002** | **P < 0.001** |
| Gabaergic | ♂ | Control | 100 | 77.1 | 81.0 | 30.0 | 95.0 | 72.0 | 87.0 | 59.0 | 88.0 | 149.0 | 12.2 | 1.2 |  |  |
|  |  | Mutant | 100 | 64.5 | 65.5 | 32.0 | 81.0 | 60.0 | 70.0 | 56.0 | 74.0 | 66.1 | 8.1 | 0.8 | **P < 0.0001** | **P < 0.001** |
|  | ♀ | Control | 100 | 67.0 | 66.0 | 26.0 | 93.0 | 59.0 | 78.0 | 50.0 | 83.0 | 184.7 | 13.6 | 1.4 |  |  |
|  |  | Mutant | 100 | 60.3 | 61.0 | 35.0 | 79.0 | 55.0 | 66.0 | 46.0 | 74.0 | 104.8 | 10.2 | 1.0 | **P < 0.0001** | **P < 0.001** |

Full description of genotypes is given in the Materials and Methods section. Significant P-values are in bold case.
